# DNA Repair Enzyme Regulation Strategy for Enhanced Pancreatic Neuroendocrine Tumor Therapy via Targeting siRNA-Lipid Nanoparticles

**DOI:** 10.1021/acsnano.5c21452

**Published:** 2026-04-01

**Authors:** Fei Wang, Yan Li, Junfeng Xu, Wei Tang, Xiaowu Xu, Xin Lou, Desheng Jing, Guixiong Fan, Yi Qin, Jie Chen, Xianjun Yu, Weibo Cai, Zhongmin Tang, Shunrong Ji

**Affiliations:** † Department of Pancreatic Surgery, 89667 Fudan University Shanghai Cancer Center, Shanghai 200032, China; ‡ Department of Oncology, Shanghai Medical College, Fudan University, Shanghai 200032, China; § Shanghai Pancreatic Cancer Institute, Shanghai 200032, China; ∥ Shanghai Key Laboratory of Precision Medicine for Pancreatic Cancer, Shanghai 200032, China; ⊥ Pancreatic Cancer Institute, Fudan University, Shanghai 200032, China; # Department of Cardiology, Shanghai Tenth People’s Hospital, 12476 Tongji University, School of Medicine, Shanghai 200072, P. R. China; ¶ Shanghai Frontiers Science Center of Nanocatalytic Medicine, School of Medicine, Tongji University, Shanghai 200072, P. R. China; ∇ Department of Diagnostic Radiology, 89667 Fudan University Shanghai Cancer Center, Shanghai 200032, China; ○ Center for Neuroendocrine Tumors, Fudan University Shanghai Cancer Center, Shanghai 200032, China; ⧫ Departments of Radiology and Medical Physics, University of Wisconsin, Madison 53705, Wisconsin, United States

**Keywords:** lipid nanoparticle, siRNA, DNA repair enzyme, pancreatic neuroendocrine tumor, temozolomide

## Abstract

Pancreatic neuroendocrine tumors (panNETs) originate from neuroendocrine cells with high rates of metastasis, rendering many patients ineligible for surgical resection. The first-line chemotherapeutic agent Temozolomide (TMZ) for metastatic panNETs faces challenges related to resistance, primarily mediated by the DNA repair enzyme O^6^-methylguanine-DNA methyltransferase (MGMT). This resistance limits the long-term efficacy of TMZ in many patients. To overcome these challenges, we developed the lipid nanoparticles (LNPs) modified with somatostatin receptors (SSTRs) targeting peptide of Octreotide to codeliver the TMZ and MGMT-siRNA (LOTR) to improve the therapeutic efficacy of TMZ via inhibiting MGMT-mediated resistance and also reducing systemic toxicity caused by TMZ. The in vitro and in vivo results demonstrated that the LOTR system significantly sensitized the tumor response to TMZ, lowered drug resistance, and reduced off-target effects, offering a promising approach for the treatment of advanced panNETs.

## Graphical abstract

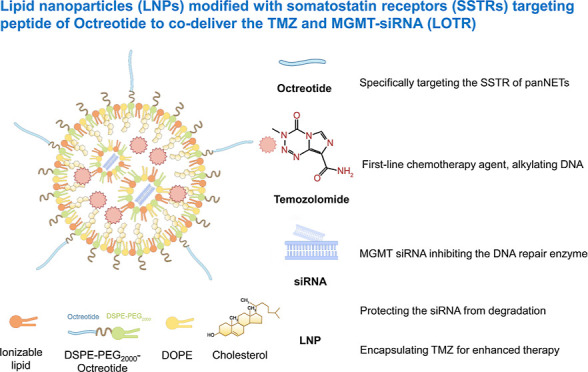

## Background of LOTR

As the major well-differentiated subtype of pancreatic neuroendocrine neoplasms (graded 1–3 by the 2019 WHO classification), pancreatic neuroendocrine tumors exhibit considerable morbidity and a higher malignant risk than commonly assumed.^1–3^ Many panNETs are found to be advanced at initial diagnosis, often with distant metastases, particularly to the liver, leading to a median overall survival (OS) of 18 months at distant metastases and a five-year OS of 53.59% for stage IV.^1,4,5^ Surgical resection remains the primary choice for panNET treatment. However, recurrence still occurs approximately 20–40% in resected patients, with peak rates observed around two years postsurgery.^6–8^ For patients with advanced or extensive metastasis who are unsuitable for surgery, Temozolomide is the standard first-line chemotherapy agent, which could disrupt essential cellular processes by alkylating DNA, leading to impaired replication and cell death.

However, a significant number of panNET patients do not respond to TMZ effectively, with an objective response rate (ORR) of 34%, and prolonged TMZ treatment often results in TMZ resistance.^9^ The primary mechanism of resistance involves DNA repair pathways, with MGMT playing a critical role as it would repair DNA lesions caused by TMZ.^10–12^ In glioblastomas, MGMT promoter methylation, which suppresses MGMT expression, has been associated with better responses to TMZ, making it a routine reference for treatment strategies.^11^

In panNETs, clinical studies have shown that combining TMZ with capecitabine (the CAPTEM regimen) significantly improves progression-free survival (PFS) in advanced panNET patients compared to TMZ monotherapy (22.7 months vs 14.4 months; hazard ratio = 0.58, ***P*** = 0.022) and MGMT deficiency has been associated with better ORR (***P*** = 0.04).^13^ However, MGMT promoter methylation is not clearly related to MGMT expression in panNET patients, and the precise role of MGMT in panNETs remains unclear. Our previous research has demonstrated that MGMT not only contributed to TMZ resistance but also acted as an oncogene, reducing the therapeutic efficacy of TMZ in patients with panNETs.^14^

Additionally, a trickier part of TMZ carries risks of toxicity and serious adverse events (AEs) are reported in 23.7% of cases.^15^ Given the risks of myelotoxicity and secondary hematologic malignancies associated with long-term TMZ use, its extended application is not usually advisible, even if initial treatment proves effective. Careful management of these risks is therefore essential.

Based on the previous research, efficient regulation of the DNA repair enzyme of MGMT could be regarded as a promising strategy to largely improve the therapeutic efficacy. Among them, RNA therapeutics, mainly including RNA interference (RNAi), mRNA, miRNA therapeutic, etc., which could modulate gene expression or interfere with post-transcriptional processes, offered novel treatments for various diseases such as genetic disorders, cancers, and viral infections, which also won the Nobel Prizes in different years.^16–20^ Moreover, the delivery system is crucial for the real clinical application of RNA-based therapeutics as RNAs are instable and easy to be degraded in vitro and in vivo.^21–23^ Among the various delivery systems, LNPs are widely recognized as one of the most promising delivery systems and the two types of LNPs for Moderna and Pfizer had helped billions of people to combat COVID-19.^24–26^ Efforts are still continuously gathered in developing more functional LNPs, including selective organs or cells targeting ability, thermostability, immunologic adjuvant and so on, which could largely benefit the disease treatment.^27–32^

Considering all the above aspects, we designed the octreotide-modified LNPs which also encapsulated the TMZ and MGMT siRNA simultaneously to realize the following goals (Scheme 1):1) the LNPs could protect the siRNA from degradation and simultaneously encapsulate TMZ for enhanced therapy; 2) the octreotide part could specifically target the SSTR of panNETs to improve the delivery efficacy; and 3) the MGMT siRNA would inhibit the DNA repair enzyme and not only largely enhance the TMZ efficacy but also inhibit the TMZ resistance. We hope to introduce the DNA repair enzyme inhibition strategy to not only benefit solving the clinical problem of panNET treatment but also enlighten the research field to introduce the RNA therapeutics to resolve the problems of clinically used drugs.

**Scheme 1:**
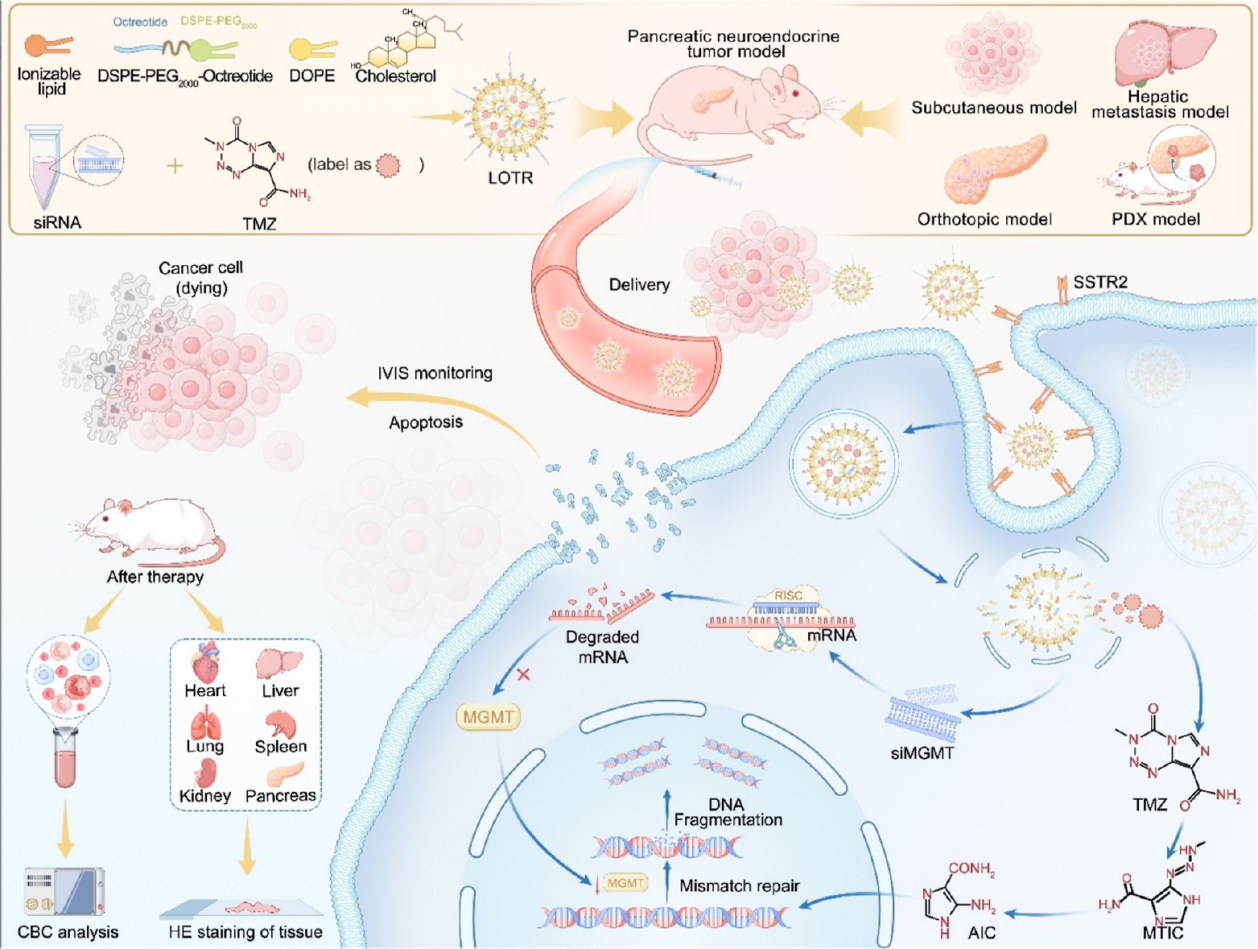
Schematic Illustration of the LOTR Nanoparticle System for panNET Therapy: Assembly, In Vivo Delivery, Therapeutic Validation, and Safety Evaluation^a^

a The LOTR nanoparticle was fabricated via the co-assembly of soybean oil-derived ionizable lipid, DSPE-PEG_2000_-Octreotide, DOPE, cholesterol, MGMT-siRNA, and Temozolomide. Therapeutic efficacy was systematically validated via in vitro functional assays and in vivo experiments in four mouse models (subcutaneous, orthotopic, liver metastasis, and patient-derived xenograft models). Following tail vein injection, LOTR crossed the vascular barrier and targeted tumor cells. Octreotide modification enables tumor-specific targeting to reduce off-target effects, and MGMT-siRNA downregulates MGMT expression to enhance the Temozolomide efficacy. For preclinical safety evaluation, complete blood count analysis and hematoxylin-eosin staining of major organs (heart, liver, spleen, lung, kidney, pancreas) were performed.

## Results

### The Synthesis and Characterizations of LOTR

As demonstrated in our previous study,^33^ we synthesized the ionizable lipid by heating the epoxidized soybean oil (ESBO) with *N*,*N*,*N*′-Triethylenediamine at a 1:7 molar ratio under solvent-free conditions at 80–90 °C for 5 days (Figure 1A). Although Figure 1A illustrated the complete reaction of all epoxy bonds, this did not guarantee uniform reactivity for all amine-containing molecules. Unreacted species were subsequently removed using a rotary evaporator at 80 °C for 4 h. The successful synthesis of the ionizable lipid was confirmed by ^1^H NMR, ^13^C NMR, and ESI-MS spectroscopy (Figures S1–S3). To formulate the LOTR, we mixed the ionizable lipid with 1,2-dioleoyl-*sn*-glycero-3-phosphoethanolamine (DOPE), DSPE-PEG_2000_-Octreotide (synthesis described in the Supporting Information, Figure S4), cholesterol, siRNA, and TMZ, forming the final LNPs as depicted in Figure 1B,C (the detailed procedure is described in the Supporting Information).

**Figure 1:**
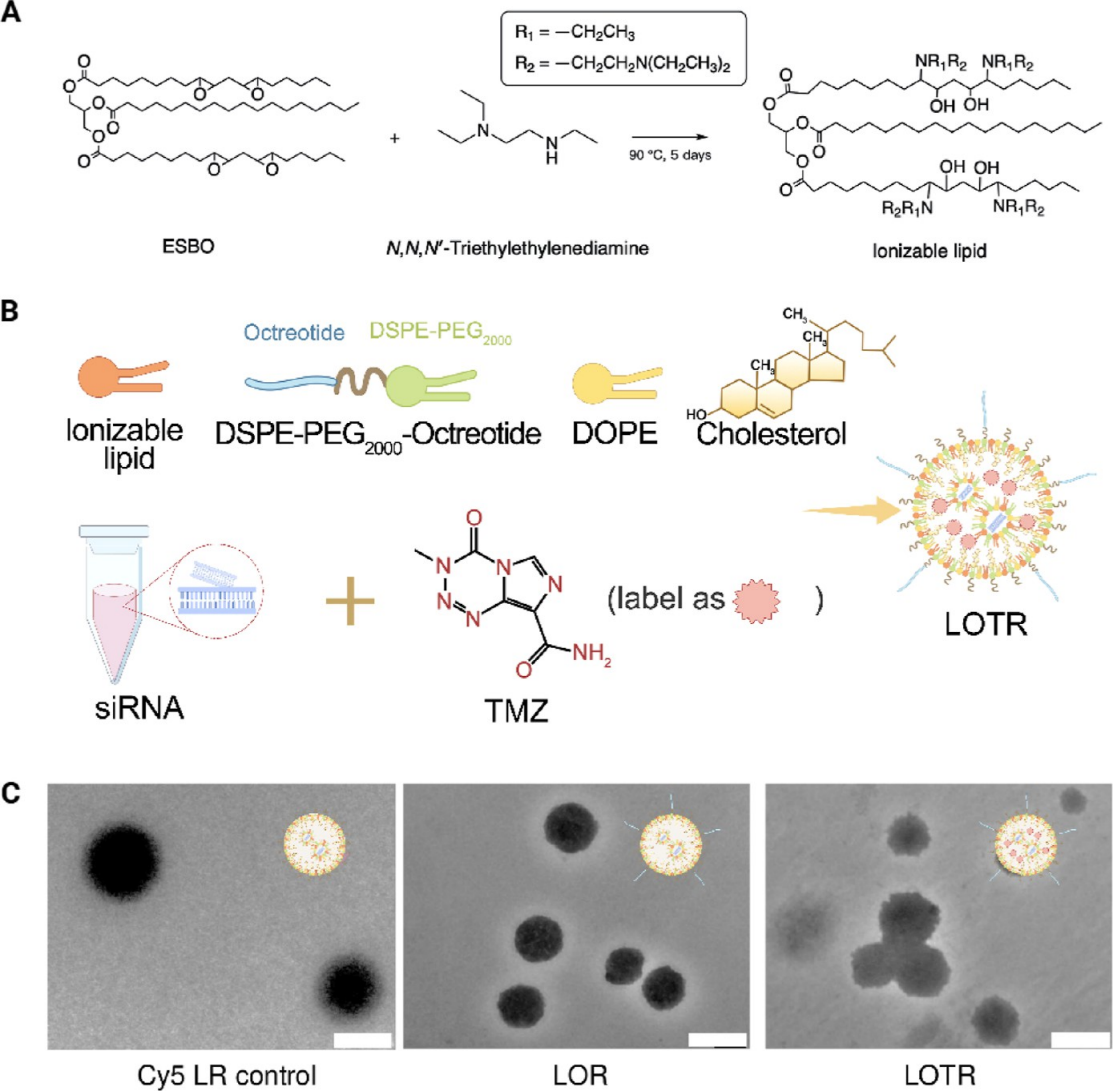
Scheme of LNPs’ formulation and characterization. (A) The synthesis process of the used soybean oil-derived ionizable lipid. (B) Schematic diagram of the LOTR assembly process, highlighting the incorporation of ionizable lipid, DSPE-PEG2000-Octreotide, DOPE, and cholesterol with siRNA and TMZ to form the final LOTR. (C) TEM images of the LNPs; Cy5 LR control: LNPs with Cy5-labeled control siRNA encapsulated; LOR: LNPs with octreotide modified and MGMT-siRNA encapsulated; LOTR: LNPs with octreotide modified, TMZ and MGMT-siRNA encapsulated; Cy5 LOTR control: LNPs with octreotide modified, TMZ and Cy5-labeled control siRNA encapsulated, demonstrating uniform spherical morphology and particle size about 200 nm. Scale bar represented as 200 nm.

We first evaluated the encapsulation efficiencies of siRNA in various LNPs by preparing gradient solutions of Cy5 labeled-MGMT-siRNA and constructing standard curves based on their specific absorbance values at 650 nm (Figure S4A). The encapsulation efficiency for LNPs with Cy5-labeled control siRNA encapsulated (Cy5 LR control) was approximately 41.86%. In contrast, LOTR (with Cy5 labeled-MGMT-siRNA) demonstrated the higher siRNA encapsulation efficiency of around 69.09%, demonstrating that octreotide modification and coloading favor siRNA retention. For TMZ, whose low UV absorbance at 329 nm limits spectrophotometric accuracy, we quantified EE by HPLC and observed 76% encapsulation under standard conditions (Figure S4B,C). This results in a final drug payload ratio of approximately 2.2:1 (TMZ/siRNA) by mass within the purified nanoparticles. To understand how a positively charged, hydrophilic drug like TMZ coloads efficiently alongside negatively charged siRNA, we then held all lipid and siRNA inputs constant and incrementally increased the initial TMZ dose up to 6-fold excess. HPLC analysis showed EE rising from 76% to 86.7% before plateauing—a saturation profile indicative of passive entrapment within the nanoparticle’s aqueous and lipid domains and a finite volumetric capacity for drug loading (Figure S5). This behavior confirms that TMZ encapsulation is driven by physical sequestration rather than electrostatic binding, allowing robust codelivery without mutual interference.

Furthermore, dynamic light scattering (DLS) and zeta potential analysis were utilized to monitor the size and surface charge across different LNPs, including Cy5 LR control, LNPs with octreotide modified and MGMT siRNA encapsulated (LOR), LOTR, and LNPs with octreotide modified which also encapsulated Cy5-labeled control siRNA and TMZ (Cy5 LOTR control) (Figure S7A,B). The hydrodynamic diameters of different LNPs ranged from 197.6 to 267.2 nm, with the LOTR formulation exhibiting the smallest size at 197.6 nm. Zeta potential values varied from −34.59 mV to −27.12 mV, with the LOTR formulation showing a zeta potential of −34.52 mV. Transmission electron microscopy (TEM) images revealed that all LNPs maintained a generally spherical and uniform morphology (Figure 1C), in which the particle sizes were consistent with DLS measurements. The well-dispersed nanoparticles with minimal aggregation indicated the good dispersibility of the formulations. These results confirmed that the coencapsulation of siRNA and TMZ does not adversely affect the LNP morphology, supporting their potential suitability for codelivery applications.

To further assess the LOTR performance, we conducted an in vitro TMZ release study alongside colloidal stability measurements. Under both physiological (pH 7.4) and acidic endosomal (pH 5.0) conditions, LOTR nanoparticles exhibited a sustained, pH-independent release of TMZ, achieving ∼80% cumulative release over 100 h (Figure S8), underscoring the robustness of the lipid matrix and predicting consistent drug availability in vivo. In parallel, dynamic light scattering revealed that both hydrodynamic diameter and the polydispersity index (PDI) gradually changed over time: at 4 °C, the diameter slowly decreased and PDI increased, reaching our defined stability threshold by 100 h, whereas at room temperature these shifts occurred more rapidly, becoming pronounced after ∼72 h in both phosphate-buffered saline (PBS) and citrate buffer (pH 5.0) (Figure S8). These time-dependent alterations coincided with the later phase of drug release and reflected controlled nanoparticle reorganization rather than premature destabilization. Together, the release and stability profiles confirmed that coencapsulation of siRNA and TMZ preserved initial nanoparticle integrity and supported LOTR as a reliable platform for sustained, controlled codelivery of dual therapeutics.

### Understanding the MGMT Roles in PanNETs

Similar to our previous research, to further elucidate the role of MGMT in panNETs in this system, we developed stable cell lines using a lentiviral system.^14^ Bon-1 cells were engineered to overexpress MGMT (Bon-1 MGMT) or to contain an empty vector as a control (Bon-1 vector). Conversely, in QGP-1 cells, MGMT was silenced using shRNA (QGP-1 shMGMT), while a nontargeting shRNA served as a control (QGP-1 NC) (Figure 2A,B). Western blot analysis confirmed the successful overexpression and knockdown of MGMT in the respective cell lines. We conducted phenotypic assays to assess MGMT’s impact on tumor growth and Temozolomide (TMZ) sensitivity. Overexpression of MGMT in Bon-1 cells significantly enhanced cell proliferation of Bon-1 cells relative to control cells (Figure 2C), In contrast, silencing of MGMT in QGP-1 cells substantially suppressed cell growth relative to controls (Figure 2D). These observations indicated that MGMT promoted tumor cell proliferation in panNETs.

**Figure 2:**
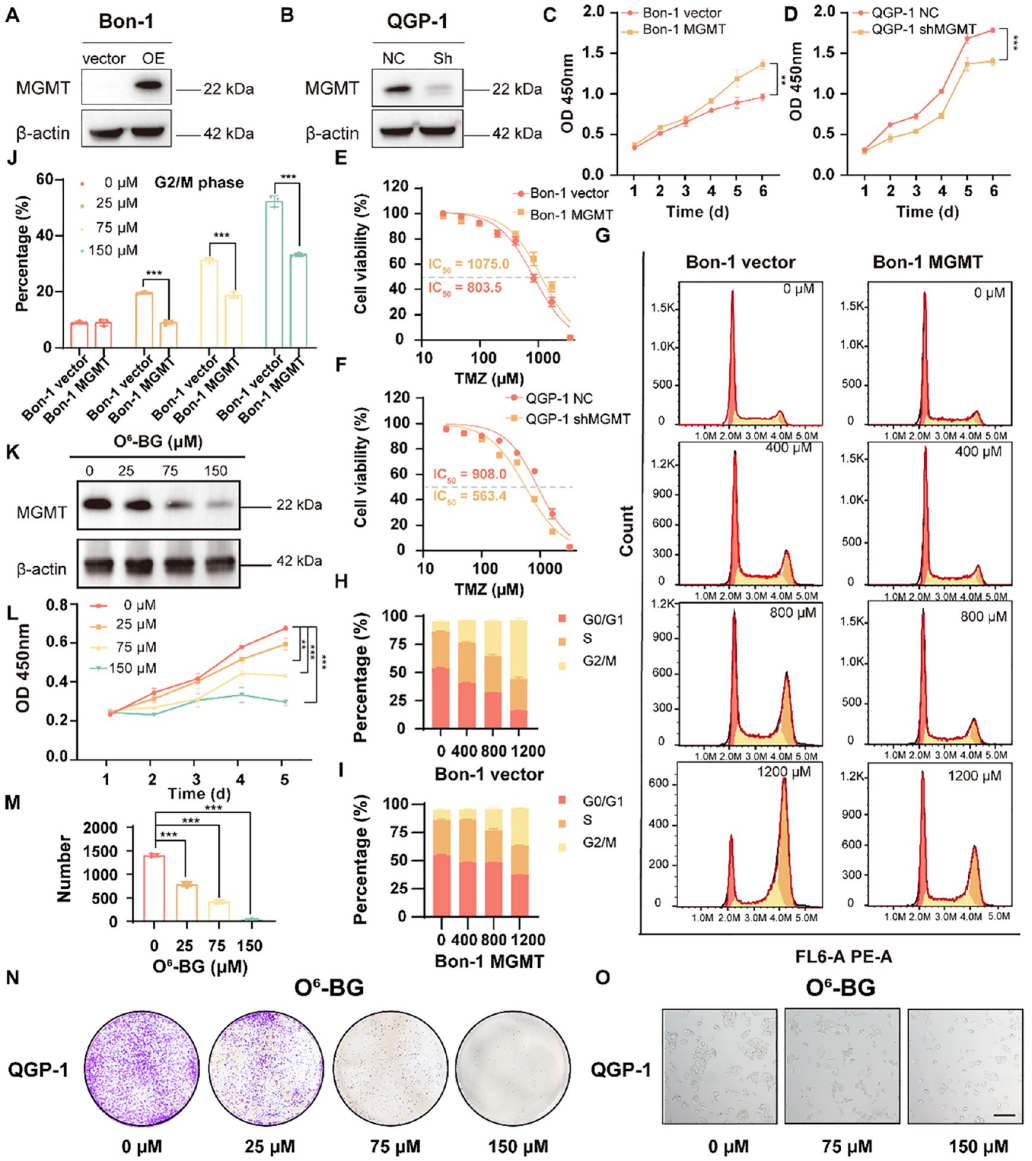
MGMT enhanced tumorigenic behavior and modulated Temozolomide sensitivity in panNET cell lines. (A,B) Western blot analysis of MGMT protein levels in Bon-1 cells overexpressing MGMT and QGP-1 cells with MGMT knockdown, compared to their respective controls. (C,D) Cell proliferation assays for Bon-1 cells with vector control or MGMT overexpression, and QGP-1 cells with control or MGMT silencing. Overexpression of MGMT significantly increased proliferation, while MGMT knockdown reduced proliferation. Approximately 3000 cells per condition were seeded in 96-well plates in triplicate and assessed using the CCK-8 assay over 6 days (mean ± SD, two-way ANOVA with the Sidak test, ** ***P*** < 0.01, *** ***P*** < 0.001, *n* = 3). (E,F) IC_50_ for TMZ in MGMT-overexpressing or silenced cells compared to controls. MGMT overexpression reduced sensitivity to TMZ, whereas MGMT silencing enhanced sensitivity. Cells were treated with increasing concentrations of TMZ for 48 h and analyzed via the CCK-8 assay (mean ± SD, *n* = 3, nonlinear regression with the model [Inhibitor] vs normalized response). (G,K) Cell cycle analysis in Bon-1 cells showing that MGMT overexpression reduced TMZ-induced G2/M arrest after 48 h of TMZ treatment. Cell cycle distribution was analyzed by flow cytometry (mean ± SD, two-way ANOVA with Sidak test, *** ***P*** < 0.001, *n* = 3). (K) Western blot analysis of QGP-1 cells treated with O^6^–BG at varying doses, demonstrating downregulation of MGMT expression. (L) Cell proliferation of QGP-1 cells treated with different doses of O^6^–BG, showing that MGMT inhibition significantly reduced proliferation (mean ± SD, two-way ANOVA with the Sidak test, ** ***P*** < 0.01, *** ***P*** < 0.001, *n* = 3). (M,N) Colony formation assay in QGP-1 cells treated with O^6^–BG. MGMT inhibition significantly decreased the colony-forming ability. Colonies were stained with crystal violet and quantified (mean ± SD, one-way ANOVA with Dunnett’s multiple comparison test, *** ***P*** < 0.001, *n* = 3). (O) Morphological alterations in QGP-1 cells treated with O^6^–BG, indicating that MGMT deficiency led to visible changes in cell morphology. Scale bar represented as 200 μm.

Next, we examined the effect of MGMT on the TMZ sensitivity. Bon-1 cells overexpressing MGMT exhibited a significant increase in TMZ resistance, as indicated by a higher IC_50_ (half maximal inhibitory concentration), compared to control cells (Figure 2E). Conversely, MGMT-silenced QGP-1 cells were more sensitive to TMZ, displaying a lower IC_50_ than control cells (Figure 2E,F and Figure S9A,B). These results suggested that MGMT expression conferred resistance to TMZ in panNET cells. To further explore the relationship between MGMT expression and cell cycle dynamics under TMZ treatment, we performed a cell cycle analysis on Bon-1 cells. Control cells exhibited a gradual G2 phase arrest with increasing concentrations of TMZ, but Bon-1 with MGMT overexpression reduced this arrest, partially rescuing the effect (Figure 2G–J, Figure S9C–F). These findings indicated that MGMT overexpression conferred resistance to TMZ-induced cell cycle arrest. Furthermore, to investigate the therapeutic potential of targeting MGMT, we treated QGP-1 cells with O^6^-benzylguanine (O^6^–BG), a potent MGMT inhibitor. Western blot analysis confirmed the downregulation of MGMT expression in a dose-dependent manner upon the treatment with O^6^–BG (Figure 2K). MGMT inhibition significantly reduced cell proliferation (Figure 2L) and colony-forming ability (Figure 2M,N) in QGP-1 cells. Higher concentrations of O^6^–BG resulted in more substantial growth inhibition and altered cell morphology (Figure 2O). These findings highlighted the therapeutic potential of targeting MGMT to overcome TMZ resistance in panNETs.

### In Vitro Therapeutic Efficacy and Mechanism Evaluation

To evaluate the biocompatibility of our ionizable lipid-based delivery system, we conducted cell proliferation assays using LR (lipid-loaded siRNA control sequence) and PBS as a control in QGP-1 cells. Over a five day period, no significant differences in cell proliferation were observed between the LR (lipid-loaded siRNA control sequence) treated group and the PBS control, indicating that the ionizable lipid only did not adversely affect normal cellular proliferation (Figure 3A). We then investigated the RNA delivering efficacy of the ionizable lipid. QGP-1 cells were treated with either LR (lipid-loaded siMGMT) or free siMGMT, and cell proliferation was assessed. Both treatments resulted in similar inhibition of cell proliferation compared with the PBS control, confirming that the loaded siRNA remains biologically active (Figure 3B). Agarose gel electrophoresis demonstrated that the ionizable lipid could protect siRNA from RNase-mediated degradation, whereas free siRNA was rapidly degraded under the same conditions (Figure 3C), indicating the crucial role of LNPs in real siRNA application. Western blot analysis further revealed that LR (lipid-loaded siMGMT) facilitated more effective delivery of siMGMT, achieving stronger knockdown of MGMT protein levels compared to free siRNA (Figure 3D).

**Figure 3:**
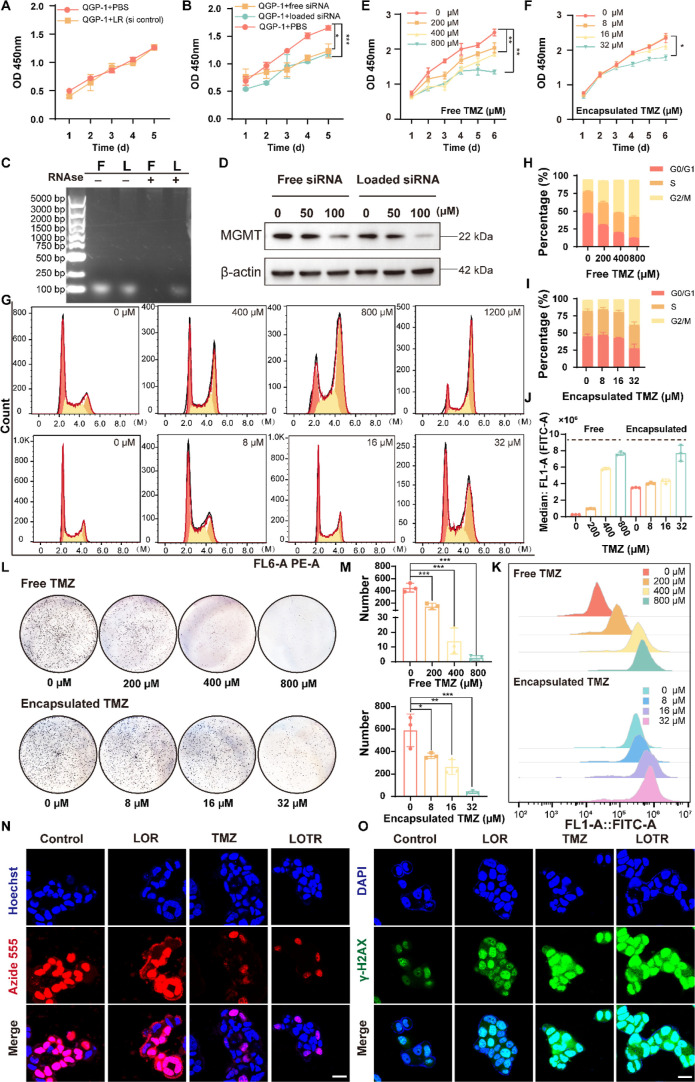
The ionizable lipid was biocompatible and enhanced siRNA delivery, reducing the required dose of TMZ. (A) Cell proliferation assay of QGP-1 cells treated with LR (loaded siRNA control sequence) or PBS. The ionizable lipid did not affect cell proliferation, as measured by the CCK-8 assay over 5 days in triplicate (mean ± SD, two-way ANOVA with the Sidak test, ***P*** = 0.19, *n* = 3). (B) Proliferation of QGP-1 cells treated with free siRNA, lipid-encapsulated siRNA, or PBS. Lipid encapsulation preserved siRNA function, as assessed by the CCK-8 assay over 5 days (mean ± SD, two-way ANOVA with the Sidak test, * ***P*** < 0.05, *** ***P*** < 0.001, *n* = 3). (C) Agarose gel electrophoresis showing that lipid-encapsulated siRNA was protected from RNase degradation, whereas free siRNA was degraded. F referred to free siRNA; L referred to lipid-encapsulated siRNA. (D) Western blot analysis of MGMT in QGP-1 cells transfected with either free or lipid-encapsulated siMGMT, demonstrating improved delivery efficiency with the lipid formulation. (E,F) Cell proliferation assay of QGP-1 cells treated with varying doses of free TMZ or lipid-encapsulated TMZ. Encapsulated TMZ at 32 μM showed efficacy comparable to free TMZ at 300–400 μM (mean ± SD, two-way ANOVA with the Sidak test, * ***P*** < 0.05, ** ***P*** < 0.01, *n* = 3). (G–I) Cell cycle analysis demonstrated that 32 μM of encapsulated TMZ induced G2/M phase arrest comparable to 200–400 μM of free TMZ. Cells were treated for 48 h and analyzed (mean ± SD, *n* = 3). (J-K) Reactive oxygen species (ROS) assay showing increased ROS production at lower doses of encapsulated TMZ compared to free TMZ. (L-M) Colony formation assay of QGP-1 cells treated with TMZ, revealing that 32 μM of encapsulated TMZ inhibited colony formation as effectively as 200–400 μM of free TMZ. Colonies were stained with crystal violet and counted (mean ± SD, one-way ANOVA with Dunnett’s multiple comparison test, *** ***P*** < 0.001, *n* = 3). (N) EdU incorporation assay, indicating that 32 μM of encapsulated TMZ in LOTR inhibited proliferation to a similar extent as 300 μM of free TMZ. Scale bar represented as 20 μm. (O) DNA damage assay using γ-H2AX staining showing that 32 μM of encapsulated TMZ in LOTR induced DNA damage comparable to 300 μM of free TMZ. Scale bar represented as 20 μm.* “Free siRNA” refers to siRNA delivered as a Lipo3000 complex (not naked siRNA), with Lipo3000 used for all in vitro siRNA transfection. Clarification for panels E–M: In these panels, “encapsulated TMZ” denotes an ionizable lipid nanoparticle coformulation containing Temozolomide and a nontargeting control siRNA.

To assess the efficacy of TMZ delivered via LNPs, we compared the antiproliferative effects of free TMZ and encapsulated TMZ (LNP-encapsulated TMZ coloaded with a nontargeting siRNA) in QGP-1 cells. Remarkably, via a DNA repair enzyme modulation strategy, 32 μM encapsulated TMZ exhibited comparable antiproliferative efficacy to 300–400 μM free TMZ, which indicated a marked enhancement in drug potency following MGMT modulation and LNP-mediated delivery (Figure 3E,F). Cell cycle analysis revealed that both free and encapsulated TMZ induced G2/M phase arrest; notably, 32 μM encapsulated TMZ elicited effects equivalent to those of 300–400 μM free TMZ (Figure 3G–I, Figure S10A–D). Reactive oxygen species (ROS) assays demonstrated that ROS production was dose-dependently elevated with both treatments and low doses of encapsulated TMZ induced ROS levels comparable to those of high doses of free TMZ (Figure 3J,K). Colony formation assays confirmed that encapsulated TMZ inhibited colony growth at significantly lower concentrations than free TMZ, with 32 μM encapsulated TMZ exerting equivalent suppressive effects on colony formation to 200–400 μM free TMZ (Figure 3L,M).5-ethynyl-2′-deoxyuridine (EdU) incorporation assays showed that both 32 μM encapsulated TMZ in LOTR and 300 μM free TMZ significantly inhibited DNA synthesis and cell proliferation to a similar extent (Figure 3N). Finally, DNA damage assessment using γ-H2AX staining indicated that 32 μM encapsulated TMZ in LOTR caused DNA double-strand breaks comparable to those induced by 300 μM free TMZ (Figure 3O).

Collectively, these findings demonstrated that the ionizable lipid-based delivery system was biocompatible and effectively combined and enhanced the therapeutic efficacy of siRNA and TMZ. The encapsulation of TMZ combined with DNA repair enzyme regulation significantly reduced the drug dosage required while maintaining potent anticancer activity, offering a promising strategy for improving therapeutic outcomes and reducing side effects associated with high-dose chemotherapy.

### In Vivo Targeting Ability and Half-Life Time Evaluation

To enhance the tumor-specific delivery of our lipid nanoparticles (LNPs), we conjugated the ionizable lipid with octreotide—a somatostatin analogue that selectively binds to somatostatin receptor 2 (SSTR2), which is overexpressed in panNET cells (Figure 4A). Immunofluorescence staining confirmed the robust surface expression of SSTR2 on QGP-1 cells, supporting the feasibility of octreotide-mediated targeted delivery (Figure 4B).

**Figure 4:**
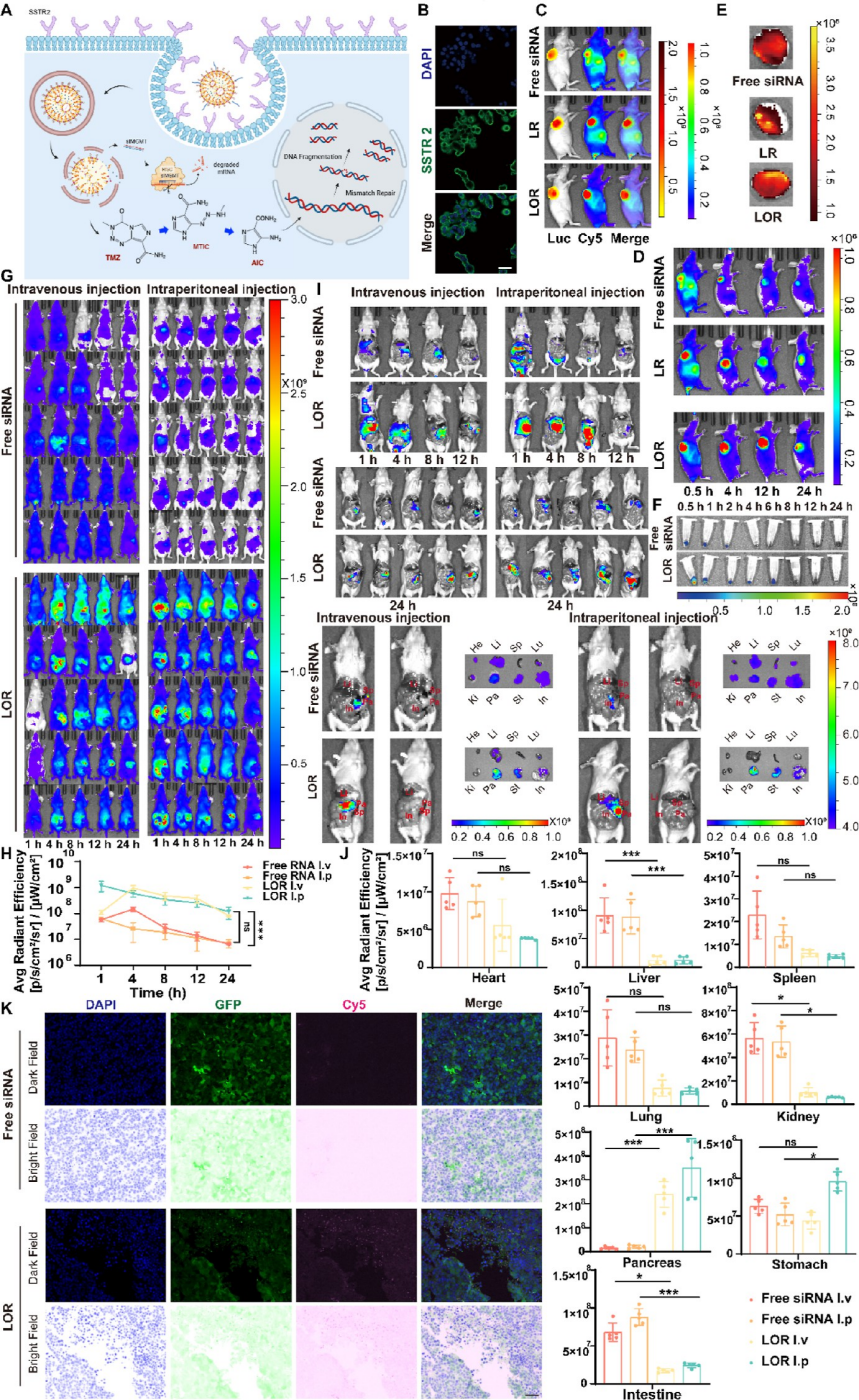
Octreotide-modified LNPs enhanced tumor targeting and prolonged siRNA circulation in vivo. (A) Schematic illustration of the mechanism by which LOTR target tumor cells expressing SSTR 2. Created in https://BioRender.com, with permission. (B) Immunofluorescence analysis confirming SSTR 2 expression on the surface of QGP-1 cells. Scale bar represented as 50 μm. (C) In vivo distribution of siRNA in mice bearing subcutaneous QGP-1 tumors, showing the highest tumor colocalization for LOR compared to LR and free siRNA. Bioluminescence (Luc) and fluorescence (Cy5) images were captured 0.5 h postinjection. Color scale indicated radiance (p/sec/cm^2^/sr) ranges (left: min = 5.00 × 10^7^, max = 2.00 × 10^9^; right: min = 5.00 × 10^6^, max = 1.00 × 10^8^). (D) Time-course fluorescence imaging demonstrating that LOR exhibited the longest circulation time compared to LR and free siRNA. Cy5 imaging was performed at 0.5, 4, 12, and 24 h postinjection. Color scale represented radiance (p/sec/cm^2^/sr) (min = 8.00 × 10^6^, max = 1.00 × 10^8^). (E) Ex vivo fluorescence imaging of harvested tumors at 24 hours postinjection, indicating higher siRNA retention in tumors from LOR-treated mice. Color scale: radiant efficiency (p/sec/cm^2^/sr/μW/cm^2^) (min = 8.64 × 10^7^, max = 3.84 × 10^8^). (F) Cy5 fluorescence imaging of blood samples collected at various time points, showing higher and more sustained levels in LOR-treated mice (0.5, 1, 2, 4, 6, 8, 12, and 24 h). Color scale referred to the range of radiant efficiency (p/sec/cm^2^/sr/μW/cm^2^) (min = 1.23 × 10^7^, max = 1.23 × 10^8^). (G) Time-course fluorescence imaging of in vivo distribution of siRNA in mice with orthotopic tumor models, demonstrating that LOR exhibited the longer circulation time compared to free siRNA. Cy5 imaging was performed at 1, 4, 8, 12, and 24 h postinjection. Color scale represented radiant efficiency (p/sec/cm^2^/sr/μW/cm^2^) (min = 5.00 × 10^7^, max = 3.00 × 10^9^). (H) Quantitative radiant efficiency of tumor growth (mean ± SD, two-way ANOVA with the Sidak test, *** ***P*** < 0.001, *n* = 5). (I) Ex vivo fluorescence imaging of the exposed abdominal cavity in mice, Cy5 imaging was performed at 1, 4, 8,12, and 24 h postinjection. Imaging of paired isolated organs for each group, including heart, liver, spleen, lung, kidney, pancreas (tumor), stomach, and intestine at 24 h postinjection. Color scale represented radiant efficiency (p/sec/cm^2^/sr/μW/cm^2^) (mice, min = 4.00 × 10^8^, max = 8.00 × 10^8^) (organs, min = 3.00 × 10^7^, max = 1.00 × 10^9^). He: heart; Li: liver; Sp: spleen; Lu: lung; *K*_i_: kidney; Pa: pancreas; St: stomach; and In: intestine. (J) Quantitative radiant efficiency of harvested organs (mean ± SD, two-way ANOVA with the Sidak test, * ***P*** < 0.05, *** ***P*** < 0.001, *n* = 5). (K) Immunofluorescence analysis of tumor tissues stained with DAPI and Cy5, showing more efficient and sustained siRNA distribution in LOR-treated tumor tissues. Tumors were scanned for DAPI, GFP, and Cy5 intensity. Scale bar: 50 μm.

To evaluate in vivo biodistribution, we employed a subcutaneous mouse model bearing luciferase-GFP-labeled QGP-1 tumors and administered free siRNA, lipid-encapsulated siRNA (LR), or octreotide-functionalized LNPs (LOR) (Figure S11A). Bioluminescence and fluorescence imaging at 0.5 h postinjection revealed superior tumor accumulation in the LOR group, followed by LR and free siRNA (Figure 4C, Figure S11B), demonstrating enhanced delivery efficiency due to octreotide modification.

Time-course fluorescence imaging (0.5, 4, 12, and 24 h postinjection) indicated that LOR achieved the longest systemic circulation, whereas free siRNA was rapidly cleared (Figure 4D, Figure S11C). Ex vivo imaging at 24 h postinjection further confirmed significantly greater tumor retention of siRNA in the LOR group (Figure 4E). Plasma analysis at multiple time points (0.5–24 h) corroborated the prolonged circulation of LOR relative to free siRNA (Figure 4F, Figure S11D).

For a more detailed assessment of nanoparticle biodistribution, we conducted in vivo imaging using orthotopic tumor models (Figure S11E). Mice received Cy5-labeled LOR or free siRNA via intravenous (IV) or intraperitoneal (IP) injection. Imaging at 1, 4, 8, 12, and 24 h postinjection showed peak tumor accumulation at 4 h (IV) and 1 h (IP) for LOR-treated mice, while free siRNA was mostly cleared by 8 h (Figure 4G,H).

Quantitative organ biodistribution analysis revealed that LOR enhanced tumor uptake by approximately 2-fold compared to that of free siRNA: IV injection yielded 16.02 × higher accumulation (free siRNA: 1.50 × 10^07^ ± 5.19 × 10^06^ vs LOR: 2.39 × 10^08^ ± 5.38 × 10^07^), and IP injection showed 17.64× higher levels (free siRNA: 1.99 × 10^07^ ± 6.84 × 10^06^ vs LOR: 3.51 × 10^08^ ± 1.22 × 10^08^). Concurrently, liver uptake was reduced by 87.09% (IV) and 86.57% (IP), indicating enhanced tumor selectivity and minimized off-target exposure (Figures 4I,J and S11F, Table S1).

Interestingly, we observed that following intraperitoneal (IP) injection of LOR, in addition to strong pancreatic tumor accumulation, a moderate Cy5 signal was also detected in the stomach. This signal was not observed in the IV-administered LOR or in any free siRNA groups. Quantitatively, the average stomach signal in the IP-LOR group was 9.58 × 10^07^ ± 1.27 × 10^07^, which—although lower than the tumor signal (3.51 × 10^08^ ± 1.22 × 10^08^)—was markedly higher than in the IV-LOR group (4.39 × 10^07^ ± 1.10 × 10^07^).

We attributed this gastric signal to several mechanistic factors: (1) the high in vivo stability and prolonged circulation of LOR particles allowed for enhanced detectability even at low exposure sites; (2) anatomical proximity of the stomach to the injection site increased local contact probability following IP administration; and (3) low-level expression of SSTR2 on gastric serosa may have facilitated weak ligand-mediated binding.^34^ Notably, this signal did not exceed that observed in tumors and was absent in other nontarget organs, suggesting that it represents a physiologically explainable secondary localization rather than an off-target effect of the targeting design.

Based on these insights, and to reduce variability and minimize nonspecific regional retention, all subsequent in vivo studies—including therapeutic efficacy and mechanistic validation—were conducted exclusively using intravenous injection. This route achieved more consistent tumor accumulation with a lower background signal, providing a more favorable tumor-to-nontumor signal ratio and improved translational relevance for clinical siRNA delivery platforms.

Immunofluorescence analysis of tumor sections from subcutaneous models confirmed a more robust and sustained distribution in the LOR group, as reflected by Cy5 fluorescence intensity (Figure 4J). Collectively, these findings demonstrated that octreotide-functionalized LNPs significantly improve siRNA delivery efficiency and tumor selectivity while prolonging systemic circulation—properties that may enhance therapeutic efficacy in panNET treatment.

### In Vivo Therapeutic Efficacy Evaluation in Various Mouse Models

To evaluate the therapeutic efficacy of the LOTR nanoparticle system, we employed multiple panNET mouse models for in vivo experiments. For the subcutaneous tumor model, mice implanted with luciferase-GFP-labeled QGP-1 cells were randomized into four experimental groups: the LOTR group, LOTR control group (loaded with nontargeting control siRNA), PBS group (negative control), and high-dose TMZ group (positive control, 40 mg/kg) (Figure 5A). The dosing regimens for each group were as follows: the LOTR and LOTR control groups received tail vein intravenous injections of 0.4 mg/kg siRNA every 3 days; the high-dose TMZ group was administered 40 mg/kg free TMZ (the TMZ dosage encapsulated in the LOTR formulation was only 0.88 mg/kg by comparison); and the PBS group was given an equal volume of PBS via the same administration route. Detailed dosage parameters for all groups are listed in Table S2. Treatment commenced on day 0, and tumor growth was monitored using an In Vivo Imaging System (IVIS) at multiple time points (Figure 5B,C). Tumor volume was measured every 4 days to assess treatment efficacy (Figure 5D). To evaluate potential systemic toxicity, mice body weights were monitored, and detailed anatomical imaging of tumors was performed for comprehensive assessment (Figure S12A,B).

**Figure 5:**
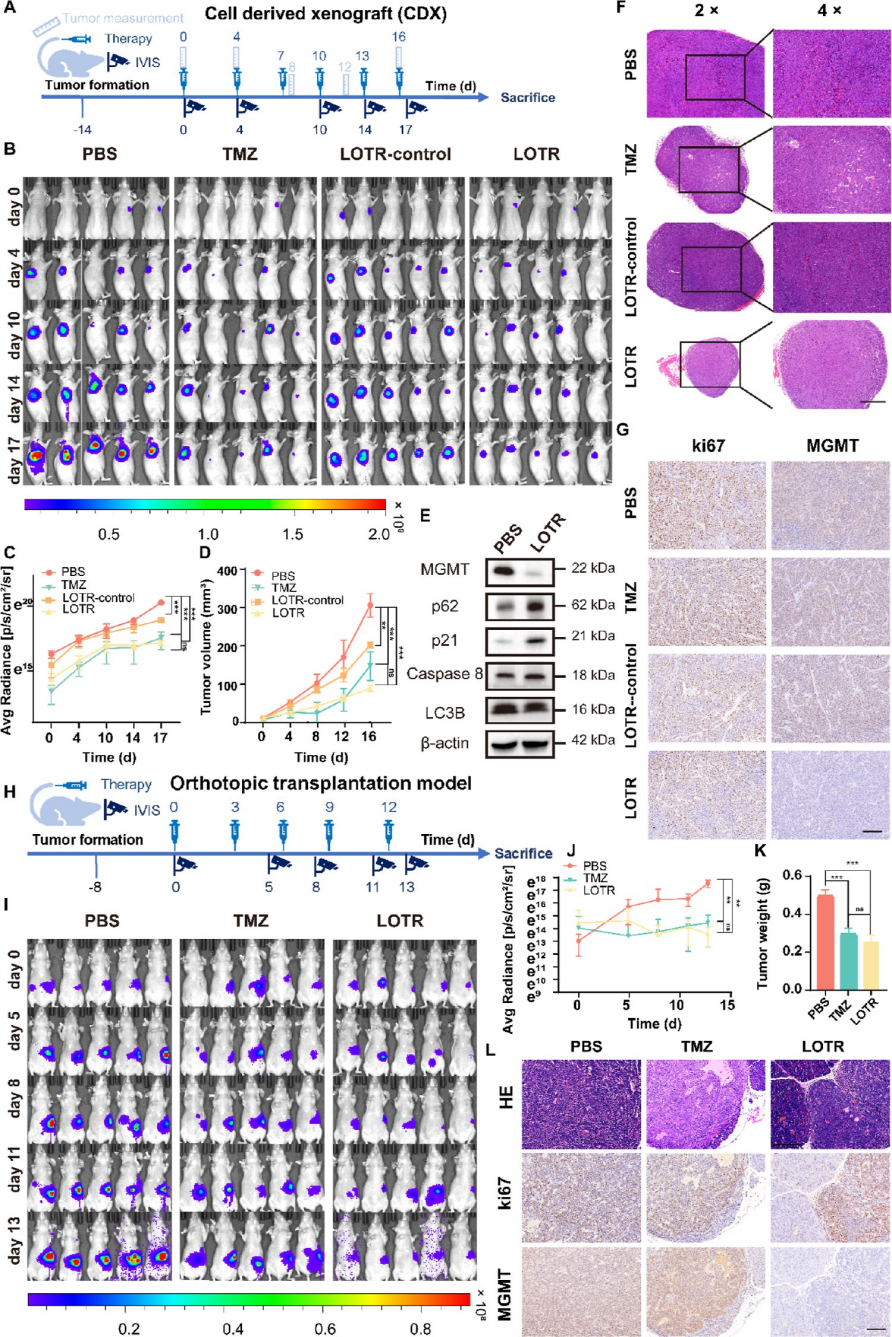
LOTR demonstrated therapeutic efficacy in subcutaneous and orthotopic panNET models. (A) Schematic diagram of the experimental design for the subcutaneous tumor model. Luc-GFP-labeled QGP-1 cells were injected subcutaneously into mice. Treatment started 14 days postinjection, and tumor growth was monitored using IVIS imaging at days 0, 4, 10, 14, and 17. (B) IVIS images showing tumor growth and treatment response over time. Color scale indicated radiance (p/sec/cm^2^/sr) (min: 1.00 × 10^7^, max: 2.00 × 10^9^). (C) Quantitative radiance analysis of tumor growth and therapeutic response (mean ± SD, two-way ANOVA with the Sidak test, *** ***P*** < 0.001, *n* = 5). (D) Tumor volume measurements over the course of the experiment (mean ± SD, two-way ANOVA with the Sidak test, ** ***P*** < 0.01, *** ***P*** < 0.001, *n* = 5). (E) Western blot analysis of tumor tissues from subcutaneous tumor models treated with PBS and LOTR, showing specific protein expression changes. (F,G) H&E and IHC staining of tumors harvested from the subcutaneous model. Scale bars: 625 μm (H&E, 4× magnification) and 200 μm (IHC). (H) Schematic of the experimental design. On day 0, 2 × 10^6^ luc-GFP-labeled QGP-1 cells were injected into the pancreas of each mouse. Treatment began 7 days postinjection, and tumor development was monitored using IVIS imaging on days 0, 5, 8, 11, and 13. (I) IVIS images showing tumor growth and treatment response over time. Color scale referred to the range of radiance (p/sec/cm^2^/sr) (min = 3.00 × 10^5^, max = 9.00 × 10^7^). (J) Radiance analysis of tumor growth and therapeutic response (mean ± SD, two-way ANOVA with the Sidak test, ** ***P*** < 0.01, *n* = 5). (K) Tumor weights at the end of the experiment (mean ± SD, one-way ANOVA with Dunnett’s multiple comparison test, *** ***P*** < 0.001, *n* = 5). (L) H&E and IHC staining of tumors harvested from the orthotopic panNET model. Scale bar: 200 μm.

The LOTR group exhibited significant tumor shrinkage compared to both the LOTR control and high-dose TMZ groups. Notably, the LOTR formulation, which combines siMGMT and TMZ, demonstrated superior antitumor activity, despite the lower TMZ dose. At the molecular level, western blot analysis of tumor tissues revealed that MGMT were significantly downregulated in the LOTR-treated group, indicating effective siRNA-mediated knockdown (Figure 5E). Additionally, there was upregulation of p62 and p21, mild upregulation of caspase-8, and downregulation of LC3B markers associated with cell cycle arrest and apoptosis (Figure S12C,D). This molecular profile suggests that the LOTR system activates multiple pathways to suppress tumor growth, impairing DNA repair and inducing programmed cell death. Furthermore, histological analysis through hematoxylin and eosin (H&E) staining and immunohistochemistry (IHC) confirmed these molecular findings (Figure 5F,G, Figure S12E). IHC staining demonstrated a marked reduction of MGMT expression in tumor tissues from the LOTR-treated group, consistent with the western blot results. Ki67 is a commonly used marker for assessing tumor proliferative activity; however, as tumor proliferation is influenced by a variety of factors, no consistent pattern of *K*_i_-67 expression was observed in our study results. These findings underscored the significance of MGMT knockdown in enhancing TMZ efficacy, as the combined delivery through the LOTR system resulted in superior antitumor activity.

To further evaluate the therapeutic efficacy of LOTR in a more clinically translatable setting, we established an orthotopic panNET model by injecting luciferase-GFP-labeled QGP-1 cells into the pancreatic parenchyma of mice (Figure 5H). The treatment regimens for this model were identical with those applied in the subcutaneous tumor model. IVIS imaging and body weight monitoring were conducted at regular intervals throughout the treatment period (Figure 5I, Figure S12F). Mice in the LOTR group exhibited significant tumor growth inhibition relative to that of all control groups (Figure 5J). And tumors harvested from the LOTR group had a significantly lower weight than those from the PBS group (Figure 5K, Figure S12G). Hematoxylin and eosin (H&E) and immunohistochemical (IHC) staining analyses confirmed these findings, revealing markedly reduced MGMT expression in tumors from the LOTR group (Figure 5L, Figure S12H). Notably, despite the substantially lower TMZ dosage encapsulated in the LOTR formulation, its tumor growth inhibition efficacy was comparable to that of the high-dose TMZ group. Furthermore, no significant body weight loss was observed in the LOTR group during the treatment, which verifies the favorable safety and biocompatibility of this therapeutic strategy.

To evaluate the effectiveness of the LOTR codelivery system in a metastatic context, we established a hepatic metastasis model using the hemispleen injection method (Figure 6A). Positron emission tomography/computed tomography (PET/CT) imaging confirmed successful model establishment and validated the rationale behind our octreotide modification (Figure 6B–D). Two weeks post-tumor cell injection, mice were treated according to the previous dosing schedule. IVIS imaging showed a marked reduction in tumor burden in the LOTR-treated group compared to that in the PBS controls (Figure 6E). No significant weight loss was observed in either treatment group (Figure S13A). Quantitative analysis of radiance and anatomical figures confirmed significant tumor suppression in the LOTR-treated mice, comparable to the high-dose TMZ group (Figure 6F, Figure S13B). H&E staining of tumor tissue further confirmed the therapeutic efficacy of LOTR in inhibiting metastatic tumor growth (Figure 6G). These results demonstrated that the LOTR system effectively targets and suppresses tumor growth in both primary and metastatic panNET models.

**Figure 6:**
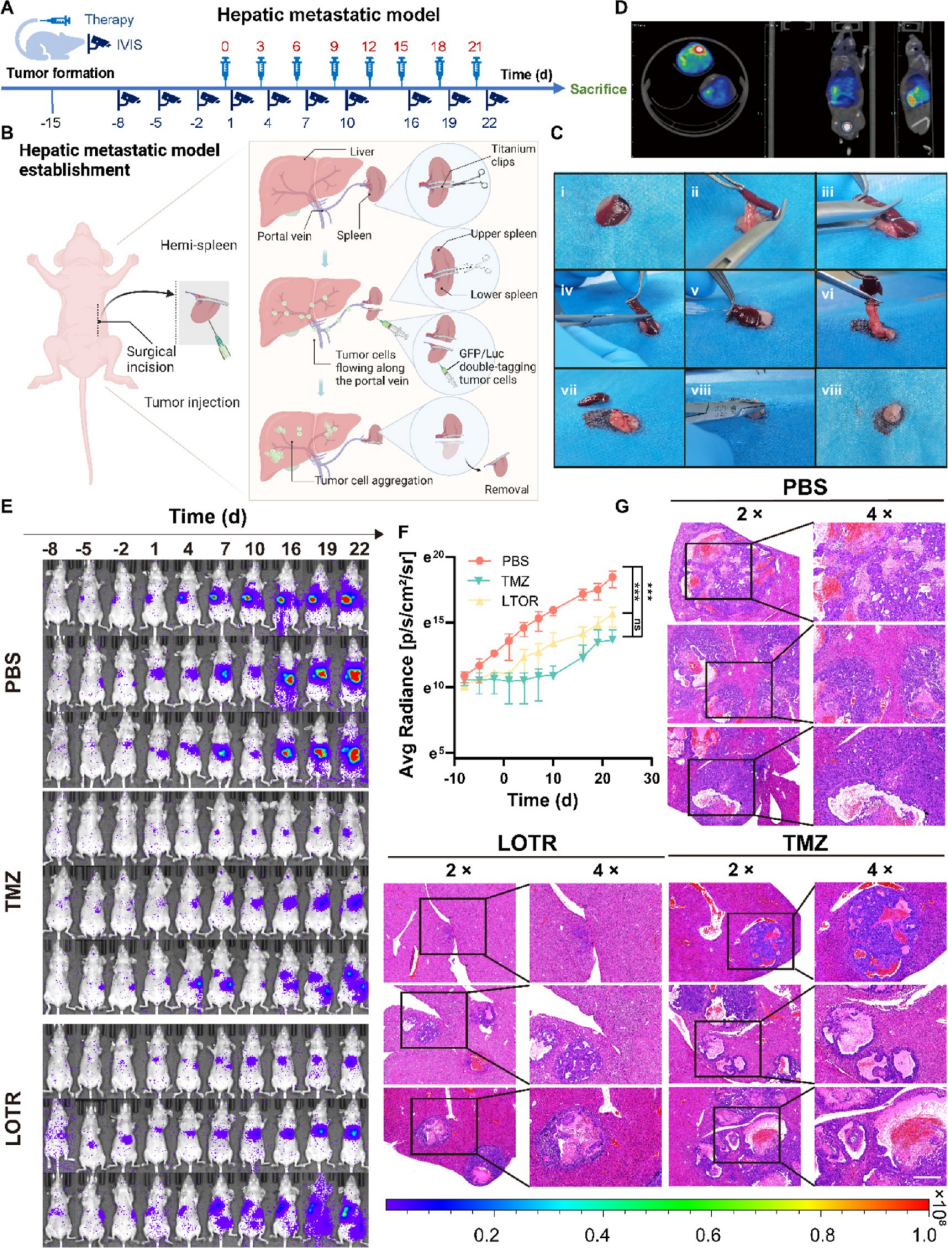
LOTR effectively inhibited tumor growth in the hepatic metastasis model of panNETs. (A) Schematic illustration of the experimental process. Luc-GFP-labeled QGP-1 cells were injected via hemispleen to establish liver metastases. Treatment started 14 days postinjection, and tumor development was tracked using IVIS imaging at multiple time points. (B) Schematic illustrating the hemispleen injection process to establish a hepatic metastasis model. After anesthetizing the mice, the spleen was divided into two parts using titanium clips. The distal spleen was injected with QGP-1 cells, and the upper spleen was retained to prevent contamination. After 10 min, the distal spleen was removed, and the abdomen was closed with sutures. Created in https://BioRender.com, with permission. (C) Surgical flowchart of hepatic metastasis model establishment. (D) PET/CT images confirming successful construction of the hepatic metastasis model. (E) IVIS images showing tumor progression and therapeutic response. Color scale indicated radiance (p/sec/cm^2^/sr) (min: 1.00 × 10^5^, max = 1.00 × 10^8^). (F) Radiance analysis of tumor growth and therapeutic response (mean ± SD, two-way ANOVA with the Sidak test, *** ***P*** < 0.001, *n* = 3). (G) H&E staining of tumors from the hepatic metastasis model. Scale bar: 625 μm (4× magnification).

In addition to the aforementioned models, we established a patient-derived xenograft (PDX) model to more faithfully recapitulate the biological complexity and intratumoral heterogeneity of human panNETs (Figure 7A,B). Tumor tissues obtained from a panNET patient were subcutaneously implanted into NSG mice, and 4 weeks postimplantation, tumor-bearing mice were randomized into six experimental groups with distinct treatment regimens: 1. PBS group (negative control); 2. High-dose TMZ group (40 mg/kg, positive control); 3. Low-dose TMZ group (0.88 mg/kg, equivalent to the TMZ dosage encapsulated in LOTR); 4. LOR group; 5. LOTR control group (octreotide-modified LNP coloaded with nontargeting control siRNA and 0.88 mg/kg TMZ, consistent with LOTR); and 6. LOTR group (0.88 mg/kg of TMZ, core experimental group).

**Figure 7:**
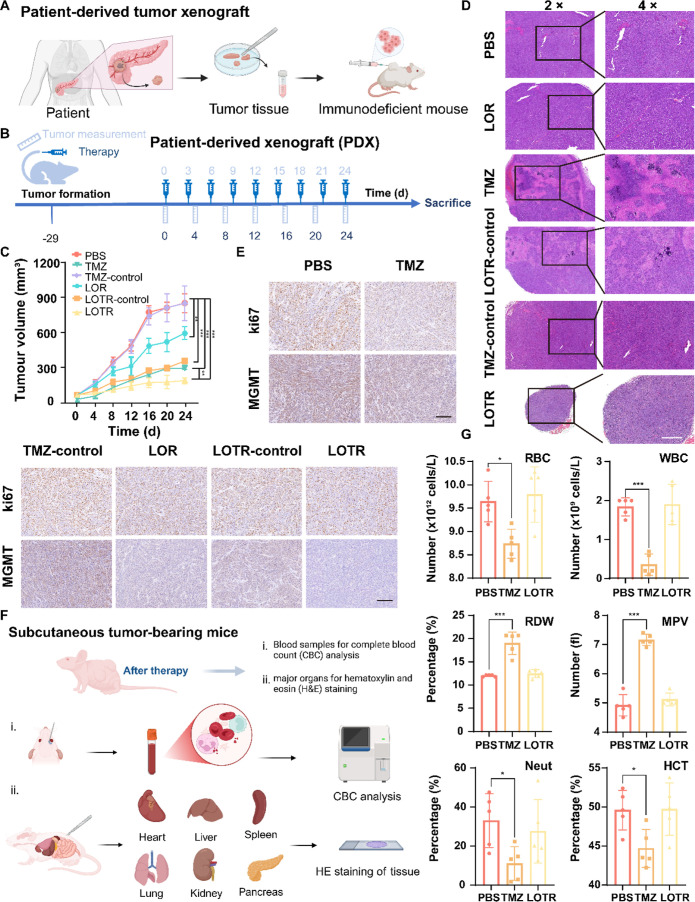
LOTR demonstrated significant antitumor efficacy in the patient-derived xenograft (PDX) model and exhibited safety and tumor-targeting specificity in vivo. (A) Workflow of PDX model establishment: patient-derived tumor tissues are collected, minced into small fragments, and then subcutaneously implanted into the flanks of immunodeficient mice. Created in https://BioRender.com, with permission. (B) Schematic diagram of the experimental design. PDX models were established, with treatment beginning 4 weeks after implantation. Tumor volumes were measured every 4 days. (C) Tumor volume measurements over the course of the experiment (mean ± SD, two-way ANOVA with the Sidak test, ** ***P*** < 0.01, *** ***P*** < 0.001, *n* = 5). (D,E) H&E and IHC staining for tumors harvested from subcutaneous and PDX models, Scale bars: 625 μm (H&E, 4× magnification) and 200 μm (IHC). (F) Schematic diagram of safety examination. Created in https://BioRender.com, with permission. (G) Complete blood count (CBC) analysis of blood samples from subcutaneous tumor models treated with PBS, high-dose TMZ, or LOTR (mean ± SD, one-way ANOVA with Dunnett’s multiple comparison test, * ***P*** < 0.05, *** ***P*** < 0.001, *n* = 5).

Tumor volume was measured every 4 days to dynamically monitor tumor growth (Figure 7C), while mouse body weight and gross anatomical analysis of excised tumors were additionally assessed (Figure S14A,B). The LOTR group exhibited the most significant tumor volume reduction compared with all other groups, which underscores the critical role of MGMT silencing in potentiating TMZ’s therapeutic efficacy. The LOTR control group—containing the same 0.88 mg/kg TMZ but nontargeting control siRNA instead of MGMT siRNA—also exerted tumor suppressive effects, albeit to a markedly lesser degree than the LOTR group. Hematoxylin and eosin (H&E) and immunohistochemical (IHC) staining analyses further validated these in vivo findings, revealing significantly decreased MGMT protein expression in tumors from the LOTR-treated group (Figure 7D,E). To investigate the in vivo safety and targeting specificity of the LOTR system, blood samples were collected from subcutaneous tumor-bearing mice for complete blood count (CBC) analysis. Major organs (heart, liver, spleen, lungs, kidneys, pancreas) were harvested, embedded in paraffin, sectioned, and stained with H&E to assess tissue integrity and potential toxicity (Figure 7F,G, Figures S14C, S15, and S16).

CBC analysis revealed that mice treated with high-dose TMZ exhibited significant hematological toxicity including decreased white blood cell (WBC) and red blood cell (RBC) counts, reduced neutrophil (Neut) percentage, and altered hematocrit (HCT) levels (Figure 7G). These changes indicate myelosuppression, a common side effect of chemotherapy leading to increased infection risk and anemia. Temozolomide, an alkylating agent commonly used in chemotherapy, exhibited notable hematological toxicity in mice.^35,36^ An increased Red Cell Distribution Width (RDW) suggested a broad distribution of red blood cell sizes, possibly reflecting stress erythropoiesis, with both mature and immature red cells entering the circulation. Elevated Mean Platelet Volume (MPV) may indicate enhanced platelet production in response to bone marrow stress. Overall, these hematological changes imply that TMZ induces significant myelosuppressive effects, with increased RDW and MPV reflecting compensatory mechanisms in response to cytotoxicity. These findings aligned with the known hematological side effects of TMZ. Previous studies, such as Terri et al., have shown that MGMT expression may correlate with the risk of myelotoxicity in chemotherapy.^35^ In contrast, the LOTR-treated group showed hematological parameters comparable to those of the PBS control, suggesting reduced systemic toxicity.

Histopathological evaluations of organ tissues in the TMZ treatment group revealed significant damage in the liver, spleen, lungs, kidneys, and pancreas, while cardiac tissue remained intact across all groups, exhibiting no discernible damage. The liver tissue in the TMZ group demonstrated substantial swelling and congestion, in contrast to that in the LOTR group, which exhibited milder hepatic injury. Notably, significant splenic congestion was observed in the TMZ group. In pulmonary tissues, TMZ treatment resulted in interstitial congestion and alveolar damage, while the LOTR group displayed only mild congestion. Renal tissue from the TMZ group also showed evidence of congestion, whereas the LOTR group exhibited only mild injury with PBS-treated kidneys appearing normal. Furthermore, pancreatic tissues in the TMZ group presented with swelling and congestion, while the LOTR group displayed a marked reduction in injury and no significant abnormalities were noted in the PBS group.

These findings suggested that the LOTR system not only enhances therapeutic efficacy but also significantly reduces systemic toxicity compared to high-dose TMZ treatment. The combination of regulating DNA repair enzyme via siMGMT and TMZ in the LOTR nanoparticles effectively suppressed tumor growth while minimizing adverse effects, offering a promising therapeutic strategy for panNETs.

## Discussion

In this study, we developed and explored the LOTR codelivery system to address the limitations of TMZ therapy in panNETs, specifically aiming to overcome drug resistance and minimize systemic toxicity. Our findings demonstrated that the LOTR system significantly enhances the therapeutic efficacy of TMZ by codelivering siRNA targeting MGMT, the key enzyme responsible for mediating TMZ resistance. Surface modification of the LNPs with octreotide improved tumor-specific targeting by binding to SSTR 2, which is highly expressed in panNET cells.^37,38^ This modification resulted in an enhanced accumulation of the nanoparticles within tumor tissues, as evidenced by in vivo imaging and biodistribution studies. The targeted delivery not only increased therapeutic efficacy but also reduced off-target effects, contributing to a better safety profile. Notably, the LOTR system allowed for effective TMZ delivery at lower concentrations compared to conventional therapy, therefore reducing the risk of significant organ damage. Our organ toxicity assessments and hematological analyses revealed that while high-dose TMZ treatment induced considerable myelosuppression—evidenced by reductions in WBC, RBC, and HCT levels—the LOTR-treated group exhibited a more balanced response. Hematological toxicity was reduced, as seen by improved WBC and RBC counts, and tissue integrity was better preserved. These results are consistent with previous studies reporting the hematological and organ toxicities associated with TMZ.^39–41^ Importantly, Al-Toubah et al. indicated the lower rates of organ toxicities, including hepatotoxicity and nephrotoxicity, in the combination therapy of TMZ with agents believed to downregulate MGMT, compared to TMZ monotherapy.^41^ The ability of the LOTR system to mitigate these adverse effects represents a critical advancement in TMZ-based chemotherapy for panNETs.

Despite the promising results, there are limitations to our study. While the murine models provided valuable insights into the therapeutic potential of the LOTR system, further validation is required in more complex biological systems. Organoid models derived from human tumors could offer a more accurate representation of the tumor microenvironment and allow for a more precise evaluation of the system’s efficacy and safety prior to clinical translation. Additionally, large-scale clinical trials will be essential to establish the LOTR system across diverse patient populations and to optimize dosing strategies that maximize therapeutic outcomes while minimizing side effects.

Looking forward, the versatility of the LOTR system offers exciting opportunities for further development. The codelivery of RNA and chemotherapeutics could be extended to other cancer types that exhibit resistance to traditional therapies.^42^ By modification of the nanocarrier with different targeting ligands, the system could be adapted to target other receptor-expressing tumors, broadening its therapeutic applicability. Furthermore, future studies could explore combining the LOTR system with other emerging cancer therapies, such as immune checkpoint inhibitors, to enhance its efficacy synergistically.^43,44^ Incorporating stimuli-responsive release mechanisms, triggered by factors such as pH or temperature changes in the tumor microenvironment, could further optimize drug release kinetics, ensuring maximal therapeutic impact at the tumor site.^27,45,46^

Nanomedicine codelivery systems like LOTR offer clear advantages over established therapies such as Peptide Receptor Radionuclide Therapy (PRRT) and Antibody-Drug Conjugates (ADCs). While PRRT is limited to tumors expressing specific receptors like SSTR2, and ADCs are confined to delivering single cytotoxic agents, nanocarriers provide the flexibility to deliver a wide range of therapeutics, including gene therapy agents and radiolabeled isotopes.^47–51^ This multifunctionality allows nanomedicine to target multiple aspects of tumor biology, potentially increasing the overall therapeutic efficacy. Moreover, the use of nanocarriers enhances the stability of encapsulated agents, such as siRNA, protecting them from degradation and improving their bioavailability.

## Conclusion

In summary, this study demonstrates that the LOTR codelivery system effectively enhanced therapeutic efficacy while minimizing systemic toxicity in panNETs. The codelivery of siRNA targeting MGMT alongside TMZ downregulates MGMT expression, promotes tumor cell apoptosis, and enhances chemosensitivity. The inclusion of octreotide surface modification improves tumor-specific targeting, leading to reduced off-target effects and increased therapeutic precision. Supported by multiple in vivo models, our findings indicated that the LOTR system can deliver effective antitumor therapy at lower doses, reducing the risks of organ damage commonly associated with traditional high-dose TMZ treatment.

While further validation was necessary, particularly in human-derived organoid models and clinical trials, this study laid the groundwork for future applications of nanomedicine-based codelivery systems in panNETs and potentially other malignancies. By integrating innovative nanocarrier designs and RNA-based therapies, the LOTR represents a promising step forward in personalized, targeted cancer treatment.

## Methods

### Ethical Approval and Informed Consent

All animal experiments were performed following the protocols evaluated and approved by the Institutional Animal Care and Use Committee (IACUC) of Fudan University (FUSCC-IACUC-2024255, FUSCC-IACUC-2024254, FUSCC-IACUC-2024253, FUSCC-IACUC-2024252, FUSCC-IACUC-2023667, FUSCC-IACUC-2023644, FUSCC-IACUC-2023643, FUSCC-IACUC-2023599, and FUSCC-IACUC-2023527). Human tissue samples for the PDX model were obtained with informed consent from the patient, following guidelines set by the Clinical Research Ethics Committee of the Fudan University Shanghai Cancer Center (2105235-9).

Further details regarding the methods can be found in the Supporting Information.

## Supplementary Material



## Notes

Complete extended experimental methods and operation protocols not included in the main Methods section; supplementary characterization, cellular activity, in vivo efficacy, and biosafety evaluation data corresponding to the Results/Discussion section of the main manuscript; quantitative organ distribution analysis at 24 h postinjection, details of component dosages used in murine models, abbreviations employed in the text, and the component composition of the LNP delivery systems; and full citations for the literature cited only in the Supporting Information files (PDF)
