# Supplementary material for: DNA Repair Enzyme Regulation Strategy for Enhanced Pancreatic Neuroendocrine Tumor Therapy via Targeting siRNA-Lipid Nanoparticles
Source: ACS Nano. 2026 Apr 1;20(14):11108–25. doi: 10.1021/acsnano.5c21452 (PMC13085844; doi:10.1021/acsnano.5c21452)
Supplement: Supplementary file 1 [file nn5c21452_si_001.pdf]

Supporting Information

DNA repair enzyme regulation strategy for enhanced pancreatic neuroendocrine tumor therapy *via* targeting siRNA-lipid nanoparticles

Fei Wang[1,2,3,4,5‡], *Yan Li*[6‡], *Junfeng Xu*[1,2,3,4,5‡], *Wei Tang*[2,8‡], *Xiaowu Xu*[1,2,3,4,5], *Xin Lou*[1,2,3,4,5], *Desheng Jing*[1,2,3,4,5], *Guixiong Fan*[1,2,3,4,5], *Yi Qin*[1,2,3,4,5], *Jie Chen*[9], *Xianjun Yu*[1,2,3,4,5*], *Weibo Cai*[10*], *Zhongmin Tang*[6,7*], *Shunrong Ji*[1,2,3,4,5*]

1 Department of Pancreatic Surgery, Fudan University Shanghai Cancer Center, Shanghai 200032, China；

2 Department of Oncology, Shanghai Medical College, Fudan University, Shanghai 200032, China；

3 Shanghai Pancreatic Cancer Institute, Shanghai 200032, China；

4 Shanghai Key Laboratory of Precision Medicine for Pancreatic Cancer, Shanghai 200032, China；

5 Pancreatic Cancer Institute, Fudan University, Shanghai 200032, China；

6 Department of Cardiology, Shanghai Tenth People's Hospital, Tongji University, School of Medicine, Shanghai, 200072, P. R. China；

7 Shanghai Frontiers Science Center of Nanocatalytic Medicine, School of Medicine, Tongji University, Shanghai, 200072, P. R. China；

8 Department of Diagnostic Radiology, Fudan University Shanghai Cancer Center, Shanghai 200032, China;

9 Center for Neuroendocrine Tumors, Fudan University Shanghai Cancer Center, Shanghai 200032, China；

10 Departments of Radiology and Medical Physics, University of Wisconsin, Madison, WI, 53705, USA.

This file includes:

Supporting Information text (Experimental Methods)
Figure S1 to S16
Table S1 to S4
Supporting Information references

Supporting Information Text

Experimental Methods

Synthesis of the ionizable lipid

The ionizable lipid was synthesized by heating epoxidized soybean oil (ESBO) with N,N,N′-Triethylenediamine under solvent-free conditions, as documented in our previous work. [1] The molar ratio of ESBO epoxy groups to the amine (-NH-) groups was maintained at 1:7. The reaction mixture was heated at 80–90 °C for five days. After the reaction, unreacted starting materials were removed *via* rotary evaporation at 80 °C for 4 hours.The ESBO used contained an average of four epoxy groups available for reaction. It should be noted that the illustrative reaction scheme (**Figure 1A**) represents an idealized outcome; complete reaction of all epoxy bonds was not always achieved, potentially due to steric hindrance from both the ESBO and the amine structures. The successful synthesis and covalent attachment *via* the ring-opening reaction were confirmed by ^{1}H Nuclear Magnetic Resonance (NMR), ^{13}C NMR, and Electrospray Ionization-Mass Spectrometry (ESI-MS) (**Figure S1-S3**).

Synthesis of DSPE-PEG$_{2000}$-Octreotide

DSPE-PEG$_{2000}$-Octreotide was obtained from Xi'an ruixi Biological Technology Co., Ltd. (Ruixibio™, China). According to the manufacturer's protocol, DSPE-PEG$_{2000}$-NHS was first dissolved in anhydrous N,N-dimethylformamide (DMF) and allowed to activate under gentle stirring. Octreotide and triethylamine (TEA) were then added to the activated polymer solution, and the conjugation reaction was carried out at room temperature for 12 h. Upon completion, the reaction mixture was transferred into a 2 kDa MWCO dialysis membrane and dialyzed against deionized water for 24 h (water replaced every 4–6 h) to remove unreacted starting materials and byproducts. The dialyzed solution was lyophilized to afford DSPE-PEG$_{2000}$-Octreotide as a white solid. The structure and purity of the conjugate were confirmed by ^{1}H NMR spectroscopy.

Synthesis of LOTR Nanoparticles and Variants

LNPs were formulated using a rapid ethanol-dilution method, which involves the controlled mixing of an ethanolic lipid solution with an acidic aqueous solution containing the therapeutic payloads.

First, individual stock solutions of the lipid components were prepared in absolute ethanol at the following concentrations: ionizable lipid (160 mg/mL; MW = 1113.99 g/mol by ESI-MS), 1,2-dioleoyl-sn-glycero-3-phosphoethanolamine (DOPE; 24 mg/mL), cholesterol (16 mg/mL), and DSPE-PEG$_{2000}$-Octreotide (8 mg/mL). A mixed lipid stock solution was then prepared by combining 200 µL of the ionizable lipid stock, 400 µL of the DOPE stock, 300 µL of the cholesterol stock, and 100 µL of the DSPE-PEG$_{2000}$-Octreotide stock. This combination corresponds to a molar ratio of Ionizable lipid:DOPE:Cholesterol:DSPE-PEG$_{2000}$-Octreotide of 50:10:38.5:1.5. The resulting solution was briefly sonicated to ensure homogeneity.

Separately, an aqueous payload solution was prepared by co-dissolving MGMT-targeting siRNA and TMZ in a 10 mM citrate buffer (pH 3.0). To enhance its solubility, TMZ was added from a concentrated stock pre-dissolved in a DMSO/citrate buffer co-solvent. For nanoparticle formation, the ethanolic lipid mixture was rapidly added to the aqueous payload solution at a 1:3 volumetric ratio (ethanol:aqueous). The formulation was designed to achieve a mass ratio of ionizable lipid to siRNA to TMZ of approximately 7:1:2. Immediately after mixing, the ethanol and aqueous phases were combined by vigorous vortexing for 30 seconds, and the resulting mixture was then incubated for 15 minutes at room temperature to facilitate self-assembly. The resulting LNP suspension was purified by dialysis (10 kDa MWCO) against phosphate-buffered saline (PBS, pH 7.4) to remove unencapsulated cargo and organic solvent. Finally, the purified LNPs were diluted with PBS to the desired working concentration for subsequent *in vitro* and *in vivo* experiments.

Control and variant LNP formulations were synthesized using the identical procedure, with the following modifications:

- **LOR:** Prepared as described for LOTR, but with the omission of TMZ from the aqueous payload solution.
- **Cy5 LOTR (control):** Prepared as described for LOTR, but substituting the MGMT-targeting siRNA with a Cy5-labeled non-targeting control siRNA at an equivalent mass.
- **Cy5 LR (control):** Prepared as described for Cy5 LOTR, but substituting the DSPE-PEG$_{2000}$-Octreotide with non-targeted DSPE-PEG$_{2000}$ in the lipid stock at an equivalent molar concentration.

Encapsulation Efficiencies (EE) Measurement of siRNA

To determine the EE of siRNA, gradient solutions of MGMT-siRNA were prepared, and their absorbance was measured at 650 nm, to construct standard curves (**Figure S1A**). Freshly prepared LNPs were subjected to dialysis to remove unencapsulated siRNA and TMZ. The concentrations of siRNA in the dialysate were quantified using the established standard curves to determine the amounts encapsulated within the LNPs. EE were calculated using the formula:

$$\mathrm{EE} = \left(\frac{\mathrm{OD_{sample}}}{\mathrm{Slope}} \times \mathrm{V_{dialysis}}\right) \div \mathrm{Amount\ of\ siRNA\ used} \times 100\%$$

where OD_{sample} is the absorbance of the sample, Slope is derived from the standard curve, and $V_{dialysis}$ is the total volume after dialysis.

Measurement of TMZ EE by HPLC

HPLC Operating Conditions

Quantitative analysis was performed on a Waters Alliance e2695 Separations Module equipped with a Waters 2998 Photodiode Array (PDA) Detector. Chromatographic separation was achieved on a C18 column maintained at 35°C. An isocratic mobile phase of methanol and 0.5% (v/v) aqueous acetic acid (30:70, v/v) was used at a flow rate of 1.1 mL/min. TMZ was detected by monitoring UV absorbance at 329 nm.

EE Determination

The EE was determined using an indirect method. Freshly prepared LNP dispersion was purified from unencapsulated drug by dialysis. During this process, the free TMZ diffused out of the dialysis bag into the external medium. This external solution (dialysate) was then collected and analyzed using the HPLC conditions described above to determine the concentration of free TMZ.

Quantification was performed using a standard calibration curve generated from a series of known TMZ concentrations ranging from 0.05 to 20 μg/mL (**Figure S4C and S4D**). The EE was calculated by relating the amount of free drug measured in the dialysate to the total amount of drug initially added to the formulation, using the following equation:

$$\mathrm{EE} = \left(\frac{\text{Peak area}}{\text{Slope}} \times V_{\text{dialysis}}\right) \div \text{Amount of TMZ used} \times 100\%$$

where Peak area is the the sample's integrated peak area, Slope is derived from the standard curve, and $V_{dialysis}$ is the total volume after dialysis.

DLS and Zeta Potential Analysis

To measure the size and zeta potential of the LNPs, the nanoparticles were diluted 100-fold with PBS. Size distribution and polydispersity index (PDI) were characterized using DLS with a Zetasizer Nano ZS instrument (Malvern Instruments, Worcestershire, UK) equipped with a He-Ne laser (λ = 632 nm). Measurements were conducted at 25 °C in disposable cuvettes to minimize multiple scattering effects. Each sample was measured in triplicate, and the average hydrodynamic diameter and PDI were reported. The zeta potential was assessed using the same Zetasizer Nano ZS instrument, determining the electrophoretic mobility of the nanoparticles in aqueous suspension. Zeta potential measurements were also performed in triplicate, and the average values were calculated.

TEM Measurement

The morphology and size of the LNPs were further examined using TEM with a JEOL JEM-2100 instrument (JEOL Ltd., Tokyo, Japan) operating at an acceleration voltage of 200 kV. Freshly prepared lipid-loaded nanoparticle solutions were subjected to dialysis to remove unencapsulated siRNA and TMZ. Subsequently, 10 μL of the dialyzed nanoparticle solution was deposited onto a carbon-coated copper grid and

allowed to incubate for 1 minute. Excess solution was gently blotted using filter paper to remove unbound particles. The grids were then stained with 1% (w/v) uranyl acetate for 1 minute to enhance contrast. TEM imaging was performed to evaluate the shape, size, and dispersion of the nanoparticles.

Methods for Drug Release Study

The *in vitro* release study was performed using Pur-A-Lyzer™ Midi Dialysis Kits (MWCO [3.5 kDa], Sigma-Aldrich) in either PBS (pH 7.4) or citrate buffer (pH 5.0). Briefly, 500 µL of the LNP dispersion was loaded into the dialysis tube, which was then submerged in a larger tube containing 12.5 mL of release buffer (1% DMSO/PBS). The setup was incubated at 25 °C with agitation at 200 RPM. At predetermined time points over 100 hours, 1 mL samples were collected from the release buffer and replaced with an equal volume of fresh buffer to maintain sink conditions. The collected samples were analyzed by HPLC to quantify the amount of released TMZ. The cumulative drug release percentage was calculated based on the initial total amount of encapsulated drug, accounting for the volume replaced at each sampling point. Each experiment was conducted in triplicate for both pH conditions.

Cell Culture

The human pancreatic neuroendocrine tumor cell line Bon-1 was kindly provided by Prof. Martyn Caplin (Royal Free Hospital, London, UK), and QGP-1 was purchased from Zhongqiao Xinzhou Biotechnology Co., Ltd. (Shanghai, China). STR analysis was used to authenticate all cell lines, which were expanded and cryopreserved in our laboratory. Bon-1 cells were cultured in DMEM/F-12 medium supplemented with 10% fetal bovine serum (FBS) and 1% penicillin-streptomycin, while QGP-1 cells were maintained in RPMI 1640 medium with 10% FBS and 1% penicillin-streptomycin. Cells were incubated at 37°C in 5% CO2. Temozolomide (TMZ; S1237) and O^6-benzylguanine (O^6-BG; S3658) were purchased from Selleck (China) and used as per manufacturer protocols.

Establishment of GFP/Luc double-tagging cell

To establish GFP/ Luc double-tagged QGP-1 cell lines, lentiviral transduction was performed using expression vectors encoding both green fluorescent protein (GFP) and firefly luciferase (Luc). The lentiviral vector containing the GFP and Luc genes was packaged by co-transfecting 293T cells with helper plasmids psPAX2 (packaging plasmid) and pMD2.G (envelope plasmid), using Lipofectamine 3000 (Life Technologies, USA). Briefly, 293T cells were seeded in 10 cm culture dishes and transfected when they reached 70–80% confluence. Transfection was performed by mixing the plasmids with Lipofectamine 3000 in Opti-MEM medium (Gibco, USA), following the manufacturer’s protocol. The transfection complexes were incubated for 20 minutes at room temperature before being added to the 293T cells. After 48 hours, the supernatants containing lentiviral particles were harvested and filtered through a 0.45 µm syringe filter to remove cell debris.

QGP-1 cells were then transduced with the collected lentivirus by adding the virus-containing supernatant directly to the cells in the presence of polybrene to enhance transduction efficiency. After 48 hours, GFP/Luc-positive cells were sorted using fluorescence-activated cell sorting (FACS). Sorted cells were expanded in culture and further validated for GFP and Luc expression by fluorescence microscopy and flow cytometry.

siRNA transfection

RNAi was performed in QGP-1 cells using Lipofectamine 3000 (Life Technologies, USA) for the delivery of siRNA targeting MGMT. QGP-1 cells were seeded in 6-well plates at a density of 2 x 10^5 cells per well in complete culture medium (RPMI 1640 supplemented with 10% fetal bovine serum) and incubated for 24 hours to reach approximately 70% confluence prior to transfection. On the day of transfection, the culture medium was replaced with Opti-MEM reduced-serum medium (Gibco, USA) to enhance transfection efficiency. Free siRNA was delivered using Lipofectamine 3000, which protects siRNA from degradation and facilitates efficient cellular internalization. Therefore, all *in vitro* gene knockdown experiments were performed with siRNA-Lipo complexes, not naked siRNA

For each transfection, siRNA (50 nM final concentration) was diluted in Opti-MEM and combined with Lipofectamine 3000 according to the manufacturer's instructions. The siRNA-Lipofectamine complexes were incubated for 20 minutes at room temperature to allow complex formation. The complexes were then added dropwise to the cells, ensuring uniform distribution across the well. Transfected cells were incubated at 37°C with 5% CO_2 for 24-48 hours, then subjected to protein analysis. Knockdown efficiency was assessed by Western blotting. To improve the stability of siRNA under *in vivo* conditions, the siRNA utilized for animal experiments was subjected to 2′-O-methyl (2′-Ome) and 5′-cholesterol (5′-Chol) modifications. Furthermore, for specific siRNA molecules requiring imaging capabilities, Cy5 fluorescent labeling was incorporated. The sequence of siMGMT: GGAAGCTGCTGAAGGTTGT.

Establishment of Lentivirus-Mediated Stable Cell Lines

Stable gene expression in Bon-1 and QGP-1 cells was achieved using lentivirus-mediated transduction. For lentivirus production, the pCDH-CMV-MCS-EF1-puro vector (System Biosciences, USA), which contains the gene of MGMT, was co-transfected with the helper plasmids psPAX2 (packaging plasmid) and pMD2.G (envelope plasmid) into HEK 293T cells using lipofectamine 3000 (Life Technologies, USA). HEK 293T cells were seeded at a density of 3 x 10^6 cells per 10 cm dish and transfected when they reached 70–80% confluence. A transfection mix was prepared by adding 6 μg of pCDH plasmid, 4.5 μg of psPAX2, and 1.5 μg of pMD2.G into Opti-MEM, followed by manufacturer’s protocol. After 20 minutes of incubation, the transfection mix was added dropwise to the 293T cells. The cells were incubated for 48

hours, and the viral supernatant was collected and filtered using a 0.45 μm filter to remove debris.

For the infection of Bon-1 and QGP-1 cells, viral supernatants were added to the cells in the presence of 8 μg/mL Polybrene to enhance infection efficiency. Cells were incubated with the virus for 24 hours before the medium was replaced with fresh culture medium. Puromycin was added to select for stable clones starting 48 hours post-infection. Cells were maintained under puromycin selection for 1–2 weeks, with medium changes every 3 days. Stable clones were confirmed by Western blotting to detect the expression of MGMT.

Agarose Gel Electrophoresis

LNP encapsulated siRNA and free siRNA were prepared following the instructions inspired by Chen *et al.* .[2] To evaluate the protection of siRNA by LNP against RNase degradation, both LNP-encapsulated siRNA and free siRNA were treated with RNase A. Briefly, 10 μg of RNase A was added to 200 μL of sample solution (either free siRNA or LNP-encapsulated siRNA) and incubated at 37°C for 30 minutes. Following RNase treatment, 0.05% sodium dodecyl sulfate (SDS) was added to the LNP-encapsulated siRNA samples to disrupt the nanoparticle structure and release the encapsulated siRNA. The samples were mixed gently by pipetting and incubated at room temperature for 5–10 minutes to ensure complete disruption of the LNP.

For agarose gel electrophoresis, a 2% agarose gel was prepared by dissolving 2 g of agarose in 100 mL of 1x Tris-acetate-EDTA (TAE) buffer, followed by heating until fully dissolved. The solution was allowed to cool to approximately 50°C, and then 4SGelred (A616697, Sangon Biotech) was added for nucleic acid staining. The gel was poured into a casting tray with a comb and allowed to solidify at room temperature for approximately 30 minutes. After SDS treatment, 10 μL of each sample was mixed with 2 μL of 6x DNA loading dye. Samples of free siRNA, post-RNase treatment, were directly mixed with loading dye without SDS treatment. For electrophoresis, 10 μL of each sample was loaded into the wells of the gel, along with an DNA Marker (B500351-0500, Sangon Biotech). The gel was run at 100 V for 30–40 minutes in 1x TAE buffer. After electrophoresis, the gel was visualized under UV light using a transilluminator. The presence of intact siRNA bands in the LNP-encapsulated samples after RNase treatment and SDS disruption indicated successful protection by the LNP.

Cell Proliferation and Colony Formation Assays

QGP-1 cells were seeded in 96-well plates at 2,500 cells per well for the proliferation assay. Cell viability was assessed using the Cell Counting Kit-8 (C0042, Beyotime) or EdU Alexa Fluor 488 Proliferation Assay Kit (C0071S, Beyotime) according to manufacturer instructions. For colony formation, cells were seeded in 6-well plates and cultured for 14 days. Colonies were fixed with 4% paraformaldehyde, stained with crystal violet, and counted under a microscope.

For cell proliferation assays, tumor cells were seeded in 96-well plates at a density of 2,500 cells per well in complete growth medium. Cells were allowed to attach overnight in a humidified incubator at 37°C with 5% CO_2. Proliferation was assessed using either the Cell Counting Kit-8 or the EdU Alexa Fluor 488 Proliferation Assay Kit, following the manufacturer's instructions. For the CCK-8 assay, 10 µL of CCK-8 reagent was added to each well and incubated for 2 hours at 37°C. Absorbance was then measured at 450 nm using a microplate reader to determine cell viability. For the EdU proliferation assay, fixation, incubation and detection according to the manufacturer's protocol. Fluorescence microscopy was used to visualize EdU-positive cells, and images were captured.

For the colony formation assay, QGP-1 cells were seeded in 6-well plates at a density of 500 cells per well and cultured in complete medium for 14 days without disturbance, allowing for colony development. The medium was replaced every 3 days. After the incubation period, colonies were washed with phosphate-buffered saline (PBS), fixed with 4% paraformaldehyde for 15 minutes at room temperature, and stained with crystal violet solution for 30 minutes. Excess stain was washed away with distilled water, and colonies were counted and calculated *via* Image J.

Western Blot

Protein extracts were prepared from cell lysates. Protein Extraction from Tumor Tissue: Tumor tissues were harvested from subcutaneous xenograft mice following euthanasia in compliance with institutional animal care and use guidelines. The excised tumors were washed with ice-cold phosphate-buffered saline (PBS) to remove residual blood and debris. The tissue samples were cut into small fragments (~1 mm^3) using sterile surgical scissors, and then placed into pre-chilled lysis tubes containing stainless steel beads. These tubes were loaded into a bead homogenizer for mechanical tissue disruption. The tissue was homogenized at 4°C for 2–3 cycles of 30 seconds each, with a 30-second rest period between cycles to prevent overheating. After homogenization, 500 µL of ice-cold radioimmunoprecipitation assay (RIPA) buffer, supplemented with protease and phosphatase inhibitors (1× concentration), was added to each tube. The samples were vortexed briefly to ensure proper mixing and then incubated on ice for 30 minutes, with intermittent vertexing every 10 minutes. Following incubation, the samples were centrifuged at 14,000 g for 20 minutes at 4°C to pellet any insoluble debris. The supernatant, containing the soluble protein, was transferred carefully to a new tube without disturbing the pellet.

Protein concentrations from cells or tissues were all measured using the bicinchoninic acid (BCA) (ZJ101, Epizyme Biotech) protein assay following the manufacturer's instructions. A standard curve was generated using bovine serum albumin (BSA) standards, and absorbance was measured at 562 nm using a microplate reader. Protein samples were diluted to equal concentrations with RIPA buffer and mixed with 5×SDS sample buffer in a 1:4 ratio. The mixtures were heated at 95°C for 5 minutes to denature the proteins. Equal amounts of protein from each sample were loaded into Super-

PAGE™Bis-Tris Gels (LK310, Epizyme) for electrophoresis and subsequently transferred to polyvinylidene fluoride (PVDF) membranes for Western blot analysis. Membranes were blocked with 5% non-fat milk in Tris-Buffered Saline with Tween-20 **(**TBST) and probed with the appropriate primary and secondary antibodies. After incubation with HRP-conjugated secondary antibodies, proteins were detected using SuperPico ECL Chemiluminescence Kit (E422-01) and visualized using a chemiluminescence imaging system. Antibodies used included MGMT (MA5-32461, Thermo Fisher Scientific), p27(A19095, ABclonal), p21(ab109520, Abcam), p62 (A19700, ABclonal), Caspase-8 (A0215, ABclonal), LC3B (A19665, ABclonal), β-actin (AC038, ABclonal) were used as the loading control.

Hematoxylin and Eosin (H&E) Staining and Immunohistochemistry (IHC)
Tumor tissues harvested from murine models were fixed in 10% neutral buffered formalin for 24 hours at room temperature. After fixation, the tissues were dehydrated through a graded ethanol series, cleared in xylene, and embedded in paraffin wax. Paraffin-embedded sections were cut to a thickness of 4 μm using a microtome and mounted onto glass slides. For Hematoxylin and Eosin (H&E) staining, sections were deparaffinized in xylene and rehydrated through graded ethanol solutions. Sections were then stained with hematoxylin for 5 minutes, rinsed in running tap water, differentiated in 1% acid alcohol, and stained with eosin for 2 minutes. The stained sections were then dehydrated in ethanol, cleared in xylene, and mounted with resinous mounting medium. Histopathological changes were examined under a light microscope, and images were captured using a high-resolution digital camera.

For immunohistochemistry, paraffin sections were similarly deparaffinized and rehydrated. Antigen retrieval was performed by heating the sections in citrate buffer (10 mM, pH 6.0) using a microwave oven for 10 minutes, followed by cooling at room temperature. After antigen retrieval, sections were blocked with 5% normal goat serum for 30 minutes to prevent non-specific binding. The sections were then incubated overnight at 4°C with the following primary antibodies: Ki67 (ab15580, Abcam, 1:200) to assess cell proliferation and MGMT (MA5-32461, Thermo Fisher Scientific, 1:100) to evaluate protein expression. After washing with PBS, sections were incubated with appropriate horseradish peroxidase (HRP)-conjugated secondary antibodies for 30 minutes at room temperature. Detection was performed using the diaminobenzidine (DAB) substrate kit, following the manufacturer's instructions. Sections were then counterstained with hematoxylin, dehydrated, and mounted with resinous mounting medium. Stained slides were imaged using microscope, and images were captured using a high-resolution digital imaging system.

Immunofluorescence Staining
Cells were seeded on confocal dishes and incubated until they reached 30-50% confluency. The samples were washed with PBS, fixed with 4% paraformaldehyde for 15 minutes, and washed three times with PBS. Permeabilization was carried out with 0.5% Triton X-100 for 10 minutes, followed by another PBS wash. Nonspecific binding

was blocked with 2% BSA for 30 minutes. Primary antibodies (SSTR2, bs-10986R) were added, and cells were incubated overnight at 4°C. After washing with PBS, secondary antibodies were applied and incubated at room temperature for 1 hour. DAPI was added for nuclear staining for 10 minutes. After washing, the samples were mounted with antifade medium and examined under a fluorescence microscope.

DNA Double-Strand Break Detection (C2035S, Beyotime): Cells were seeded in confocal dishes and treated with the following conditions after overnight recovery: 300 μM free TMZ, 32 μM TMZ in LOTR (corresponding to 96 μM siRNA), or 96 μM siRNA alone. After 48 hours, the medium was aspirated, and cells were washed with PBS. Cells were fixed with 4% paraformaldehyde for 5-15 minutes. After permeabilization and blocking, cells were incubated with γ-H2AX rabbit monoclonal antibody at 4°C overnight. The next day, secondary antibodies conjugated to Alexa Fluor 488 were applied at room temperature for 1 hour. After DAPI staining, the cells were visualized under a fluorescence microscope. γ-H2AX staining appeared as green fluorescence, while DAPI-labeled nuclei appeared blue.

EdU Proliferation Assay: Cells were previously treated similar to the γ-H2AX assay conditions. After drug treatment, 2 × EdU working solution (20 μM) was pre-warmed and added to the cells for 2 hours. After fixation and permeabilization, cells were incubated with the Click-iT reaction buffer for 30 minutes in the dark. Streptavidin-HRP and DAB staining were then applied. Cells were visualized under a fluorescence microscope, and proliferation rates were analyzed.

Flow Cytometry (FACS) Analysis

Cell Cycle Analysis (C1052, Beyotime): Pre-cooled PBS and 70% ethanol were prepared. Cells were trypsinzed, collected by centrifugation (1000g, 5 minutes), and fixed overnight in 70% ethanol. Propidium iodide (PI) staining was performed, and cell cycle distribution was analyzed *via* flow cytometry.

Reactive Oxygen Species (ROS) Detection (S0033M, Beyotime): Cells were grouped and treated as described for the EdU and γ-H2AX assays. Following drug treatment, cells were incubated with DCFH-DA (10 μM final concentration) for 20 minutes at 37°C. ROS levels were measured using a flow cytometer with excitation at 488 nm and emission at 525 nm.

Blood Sample collection

The mouse is manually restrained, with its head and body securely fixed to leave the tail fully exposed. Next, the distal end of the tail (approximately 1 cm) is disinfected with an alcohol swab to maintain sterility of the sampling site. A small 1–2 mm section of the distal tail skin is then carefully excised using sterilized fine scissors or a scalpel, taking care to avoid making an overly deep incision; for the initial blood collection, only a minimal portion of the tail skin is removed. Blood is subsequently gently expressed by massaging the tail from proximal to distal to form a blood bead at the

incision, and a 10 μL micropipette is used to quantitatively aspirate blood from this blood-beading site at the tail tip. The aspirated blood is collected into a microcapillary tube (20 μL per tube). Once the desired volume of blood has been collected, hemostasis is achieved by applying gentle and continuous pressure to the incision with sterile cotton or gauze. After confirming hemostasis, the mouse is returned to its cage, and its condition is monitored to ensure recovery. Blood collection is conducted at predetermined time points: 0.5 h, 1 h, 2 h, 4 h, 6 h, 8 h, 12 h, and 24 h.

Orbital Blood Collection: The mouse is anesthetized using sodium pentobarbital. Once the mouse is fully anesthetized, it is placed in a supine position and securely restrained on the surgical platform, ensuring that the head and body are immobilized for the procedure. The area around the eye is disinfected with an alcohol swab. Using fine forceps, the surrounding tissue is gently separated, taking care to avoid damaging adjacent facial tissues. A fine ophthalmic scissor or surgical scissor is used to carefully excise the eyeball along with a portion of the surrounding tissue. As the eyeball is removed from the socket, the rupture of the posterior venous plexus allows blood to begin flowing. The blood is collected directly into a blood collection tube containing an anticoagulant. After the required volume of blood is collected, the mouse is euthanized following standard ethical procedures.

Orthotopic panNETs model

BALB/c nude mice (8-week-old, female) were anesthetized using mixed solution of zoletil combined with serazine hydrochloride (15mg/kg) according to their weight and conditions. After ensuring the depth of anesthesia, luc-GFP-labeled QGP-1 cells (2 $\times 10^6$) were suspended in cold PBS and injected into the pancreatic parenchyma using insulin syringe. The needle was kept on the injection site for 15 s to prevent the leakage of cells. The pancreas was slowly put back into the abdominal cavity, and the cavity was sutured using a two-layer running silk suture.

Establishment of hemi-spleen injection model for panNETs with liver metastasis

The protocol of hemi-spleen injection model was originally inspired and based on Soares *et al.*. [3] Firstly, we anesthetized BALB/c-nude mice (8-week-old, female) in suitable doses using mixed solution of zoletil combined with serazine hydrochloride (15mg/kg). After checking toe pinch withdrawal reflex to ensure the state of fully anesthesia, we sterilized the skin of the left subcostal area with 70% ethanol and applied a drape to keep sterility during surgery. Then the laparotomy began and an incision in the left subcostal area was made. We gently pulled out the spleen with a cotton swab, carefully checked the blood vessels of spleen and divided spleen into two hemi-spleens after applying two titanium clips in the middle of the spleen to avoid massive bleeding. Then the upper half of spleen was put back and remained in body to prevent tumor cell contamination, the other half was injected with 2 $\times 10^6$ cells suspended in 30μl phosphate buffered saline. In an ideal setting, the injected tumor cells would flow along the vein and finally into liver, establishing microscopic hepatic metastases. Thus, it is better to observe closely whether the color of the injected half spleen and blood vessels

turned white simultaneously while injecting. After 10 minutes from injection, we removed the injected half spleen after applying clip to area of pancreas and splenic vessels to prevent massive hemorrhage and the abdominal closure was followed. In the end, we performed abdominal closure and waited mouse recovery from anesthesia. To observe and record the development of liver metastasis, we utilized the IVIS after surgery and monitored the body weight weekly. In nearly 6 weeks later, we chose the CO_2 asphyxiation to euthanize mice, performed gross anatomy, harvested liver, pancreas and spleen.

Subcutaneous Xenograft Tumor Model

For the subcutaneous tumor model, 6-week-old female BALB/c nude mice were injected with 2 $\times 10^6$ QGP-1 cells in 100 μL PBS into the subcutaneous tissue of the flank. Tumor growth was monitored every four days by measuring the tumor volume with vernier calipers. The tumor volume was calculated as (length × width^2)/2. In the end, mice were euthanized, and tumors were harvested for further analysis.

Patient-Derived Xenograft (PDX) Model

For the PDX model, pancreatic neuroendocrine tumor tissue obtained from one patient was implanted into NSG mice (6-week-old, female). The patient tissue was cut into 3 ×3 mm pieces and surgically implanted into the subcutaneous space of the mice. Tumor growth was monitored every four days using vernier calipers, and after sufficient tumor growth, the tumors were harvested. This model was used to evaluate drug efficacy and tumor development under treatment conditions.

Imaging

To acquire image sequence and measure fluorescent intensity, living image software (IVIS Imaging Systems) was utilized. PerkinElmer was utilized to capture the IVIS image of models. To access the photon flux, we drew the region of interest in the upper abdominal especially liver area, and the average radiance were used to represents the average intensity. The imaging frequency and observation duration were appropriately adjusted for different mouse models based on their distinct tumor growth rates and proliferation cycles, aiming to capture sufficient imaging data reflecting tumor growth dynamics within the critical experimental window from stable tumor establishment to the point requiring ethical intervention due to excessively high tumor fluorescence signals. Inveon (Siemense) PET/CT was utilized to capture the PET/CT image of models. The firefly luciferase intensities of implantation lesions were measured settled time point by staining with D-Luciferin firefly, potassium salt (150 mg kg^{-1}, 40902ES03, Yeasen) in an *in vivo* imaging system. The mice were sacrificed in a carbon dioxide chamber at the end of the study and subjected to further study.

Therapeutic intervention

Usually, we divided murine model into three groups: PBS, TMZ and LOTR. PBS severed as negative control, TMZ positive control. We injected LOTR, LOTR-control, LOR (0.4 mg/kg siRNA per mouse, tail intravenous injection). PBS group were given

the same volume (100 uL per mouse) as LOTR. Each mouse in TMZ group were injected treated with temozolomide (40mg/kg, tail intravenous injection). TMZ control group refers to mouse given the same amount of temozolomide as in LOTR (0.88 mg/kg, tail intravenous injection) to exclude the effect of the encapsulated temozolomide. All groups were treated every three day and monitored *via* IVIS imaging and weight measurement at settled time point. All the weights of mice would be traced since tumor therapy started.

Statistical Analysis

All experiments were conducted with at least three replicates, and data are presented as the mean ± standard deviation (SD). For comparisons between multiple groups, an ordinary one-way analysis of variance (ANOVA) was performed (one-way ANOVA with Dunnett's multiple comparison test). Post-hoc multiple comparisons were conducted where appropriate to identify significant differences between individual groups. For experiments involving repeated measures over time, a two-way ANOVA with Sidak test multiple comparisons were used to assess both the effect of time and treatment across groups. IC_{50} values were calculated using nonlinear regression with the model [Inhibitor] vs. normalized response. Statistical significance was determined at thresholds of $\boldsymbol{P} < 0.05$, $\boldsymbol{P} < 0.01$, and $\boldsymbol{P} < 0.001$ as in The New England Journal of Medicine (NEJM). All analyses were performed using GraphPad Prism 9.5.0 software.

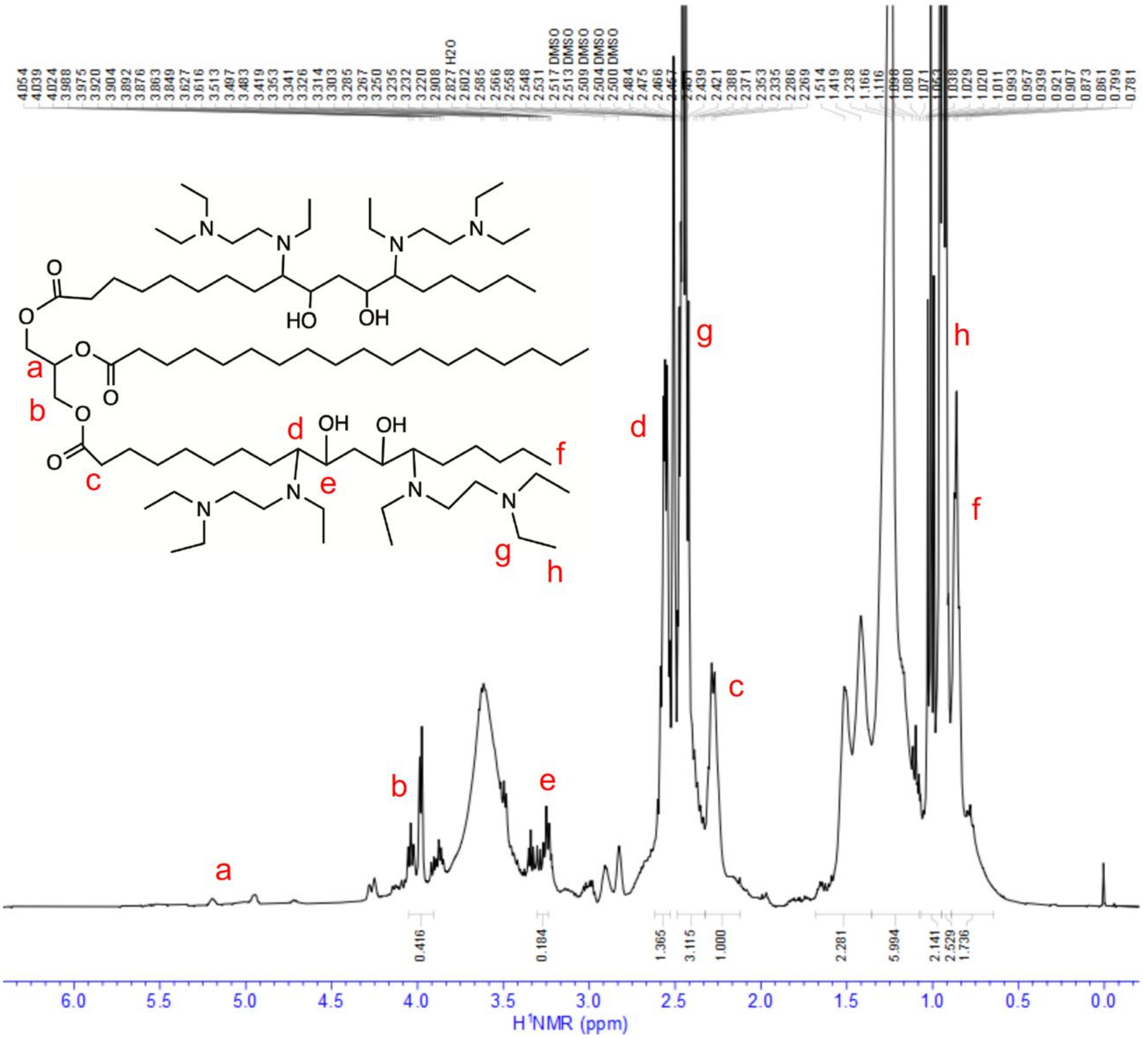

Figure S1. ^{1}H NMR (400 MHz, DMSO-d_6) spectrum of the ionizable lipid.

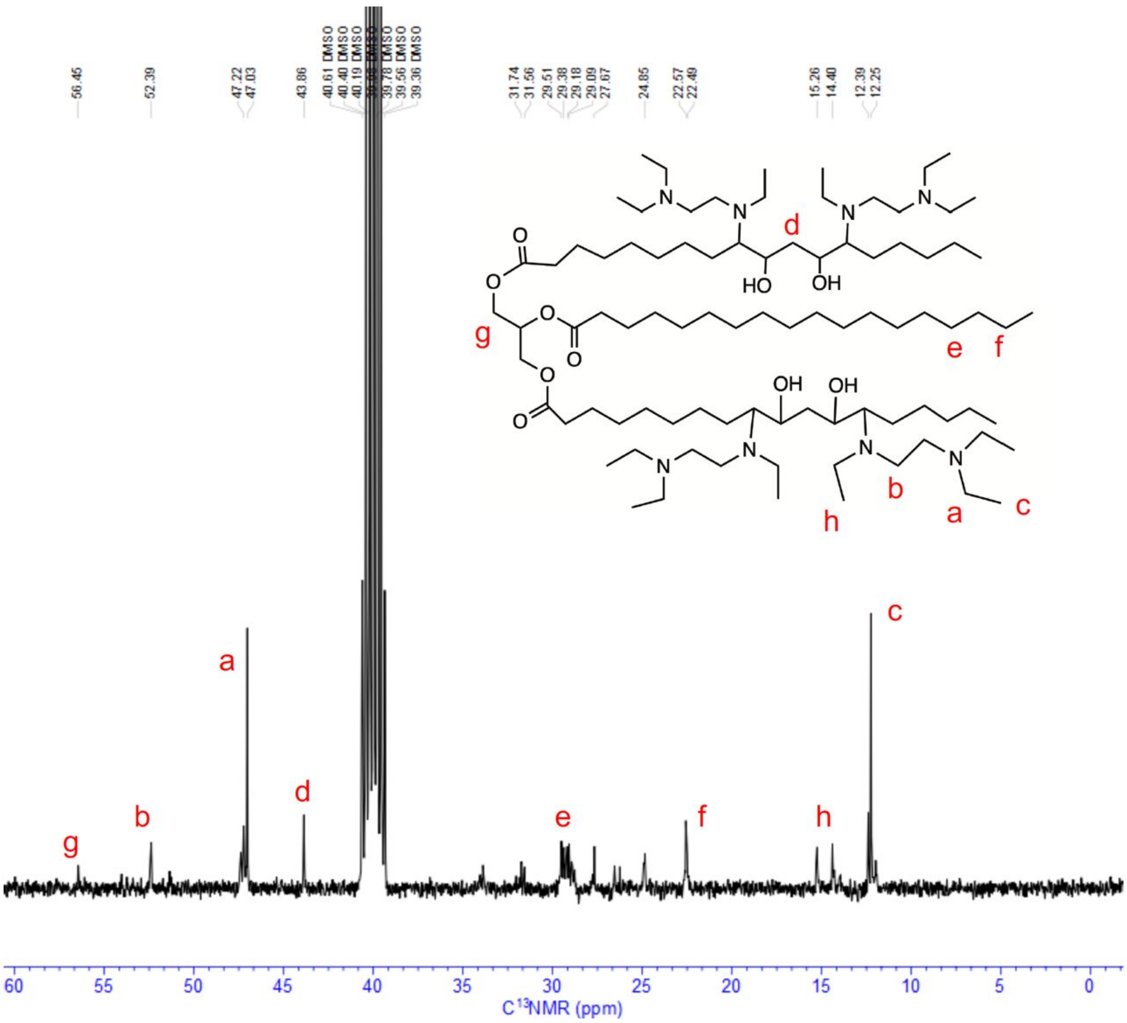

Figure S2. ^{13}C NMR (400 MHz, DMSO-d$_6$) spectrum of the ionizable lipid.

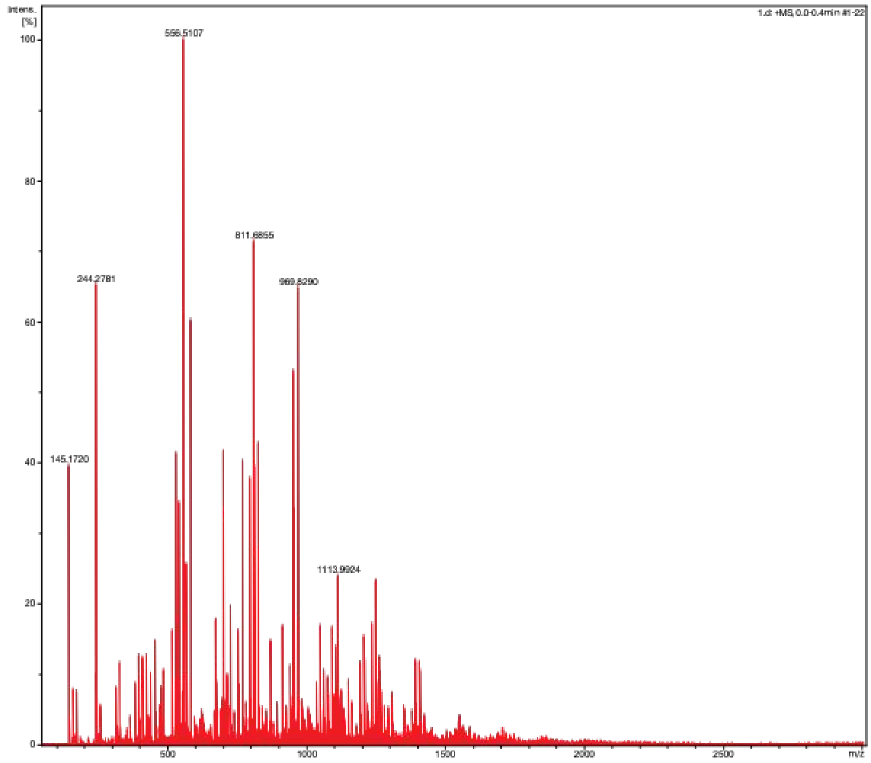

Figure S3. ESI-MS spectrum of the ionizable lipid. The spectrum displays peaks corresponding to the epoxidized soybean oil starting material (*m/z* 969.82) and products with one (*m/z* 1113.99) or more N,N,N′-Triethylenediamine moieties conjugated.

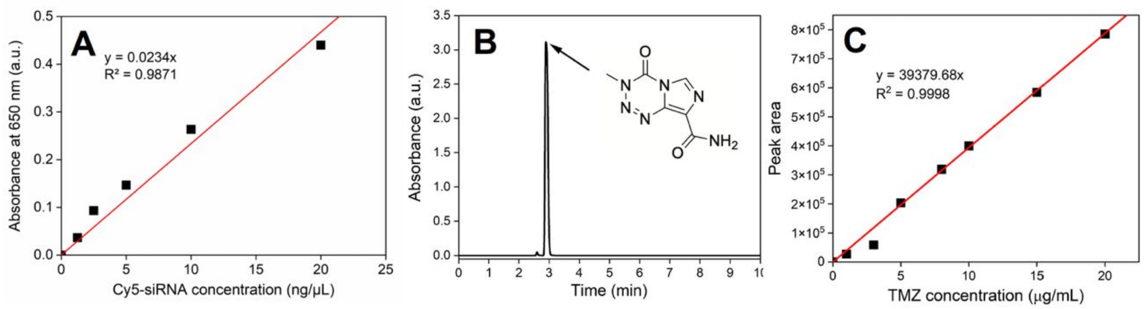

Figure S4. (A) Absorbance at 650 nm plotted against concentrations of Cy5-siRNA, with the line representing the best linear fit. (B) Trace of HPLC trace of the standard solution of TMZ. The retention time for TMZ was approximately 2.8 minutes. (C) Standard curve for TMZ quantification by HPLC. The peak integration area is plotted against standard TMZ concentrations, showing excellent linearity (R^2=0.9998).

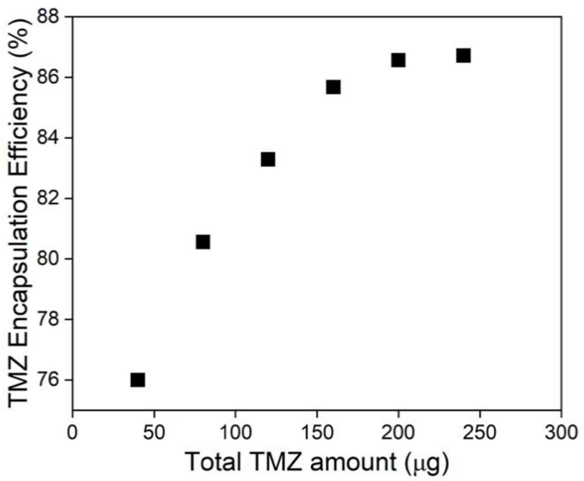

Figure S5. Effect of initial TMZ concentration on its EE%. The amount of siRNA and lipids was held constant while the initial input of TMZ was increased. The EE% of TMZ, measured by HPLC, increases before reaching a saturation point, supporting a passive entrapment model with a finite loading capacity.

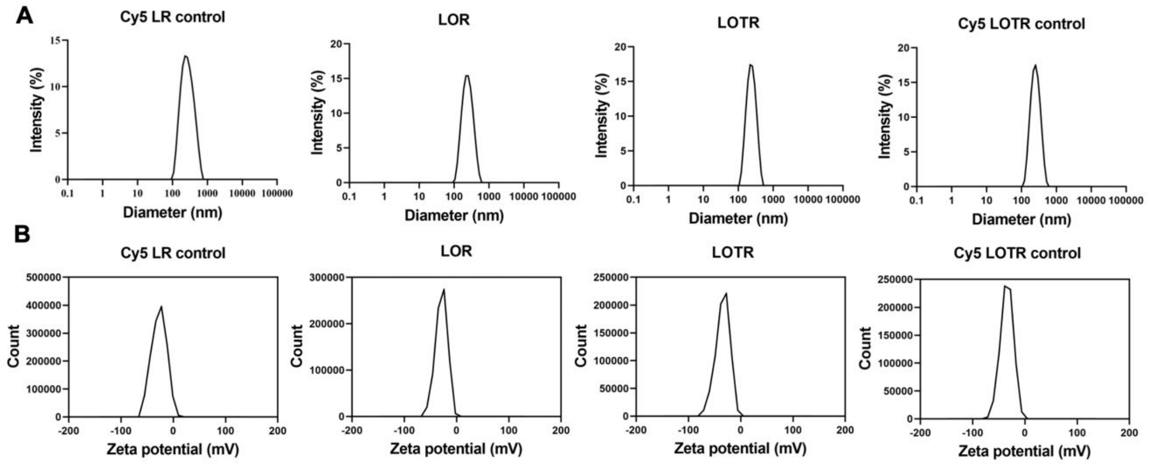

Figure S6. (A) Particle size distribution and polydispersity index (PDI) measurements of the lipid nanoparticles (LNPs). Specifically, the Cy5 LR control exhibited a particle size of 229.8 nm and a PDI of 0.521, LOR had a particle size of 239.9 nm and a PDI of 0.25, LOTR showed a particle size of 232.6 nm and a PDI of 0.132, and the Cy5 LOTR control presented a particle size of 255.1 nm with a PDI of 0.24. (B) Zeta potential and conductivity measurements of the LNPs. The Cy5 LR control displayed a zeta potential of −27.12 mV and a conductivity of 16.74 mS/cm, LOR had a zeta potential of −28.94 mV and a conductivity of 16.94 mS/cm, LOTR showed a zeta potential of −34.52 mV and a conductivity of 17.14 mS/cm, and the Cy5 LOTR control exhibited a zeta potential of −34.59 mV and a conductivity of 17.33 mS/cm.

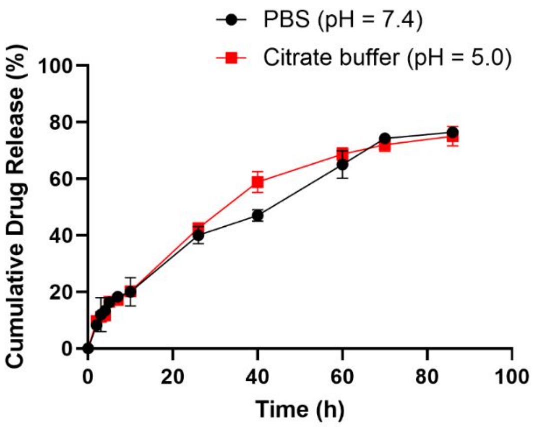

Figure S7. *In vitro* cumulative release of TMZ from LOTR nanoparticles. The graph shows the percentage of released TMZ over 100 hours at physiological pH (7.4) and acidic pH (5.0). Data are presented as mean ± SD (n = 3).

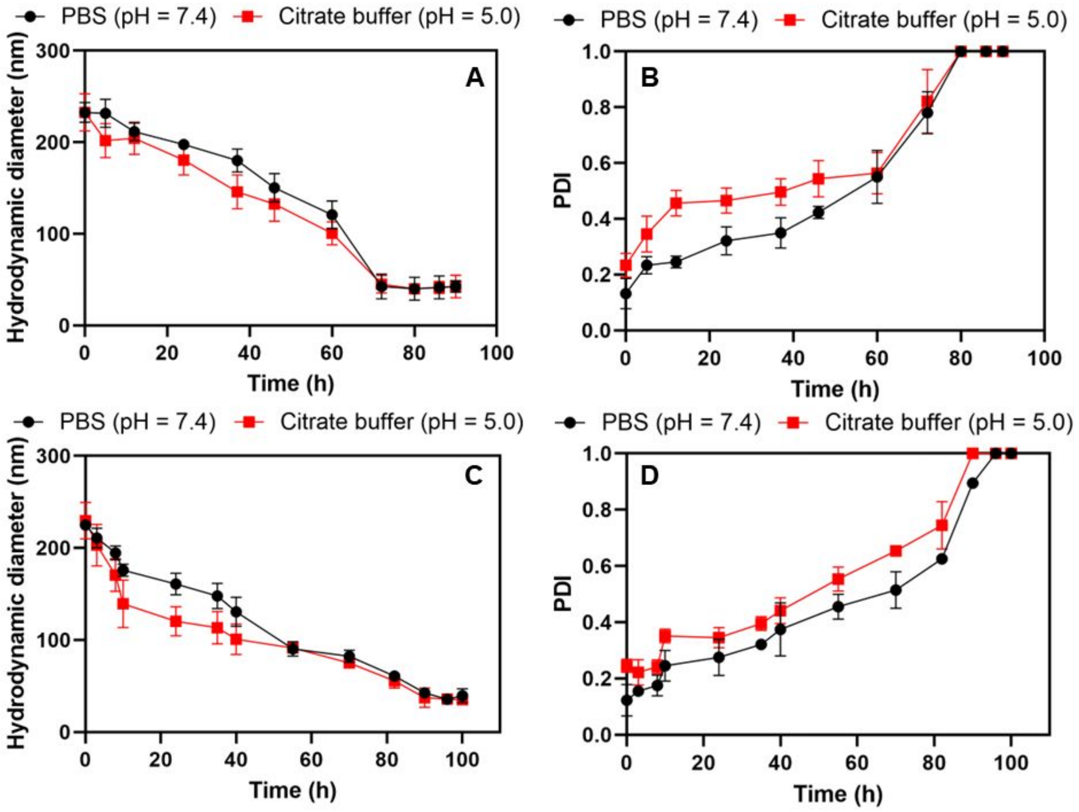

Figure S8. Changes in hydrodynamic diameter and PDI of LOTR nanoparticles over time in PBS (pH 7.4) and citrate buffer (pH 5.0) at room temperature (RT; panels A and B) and 4 °C (panels C and D). Data are presented as mean ± SD (n = 3).

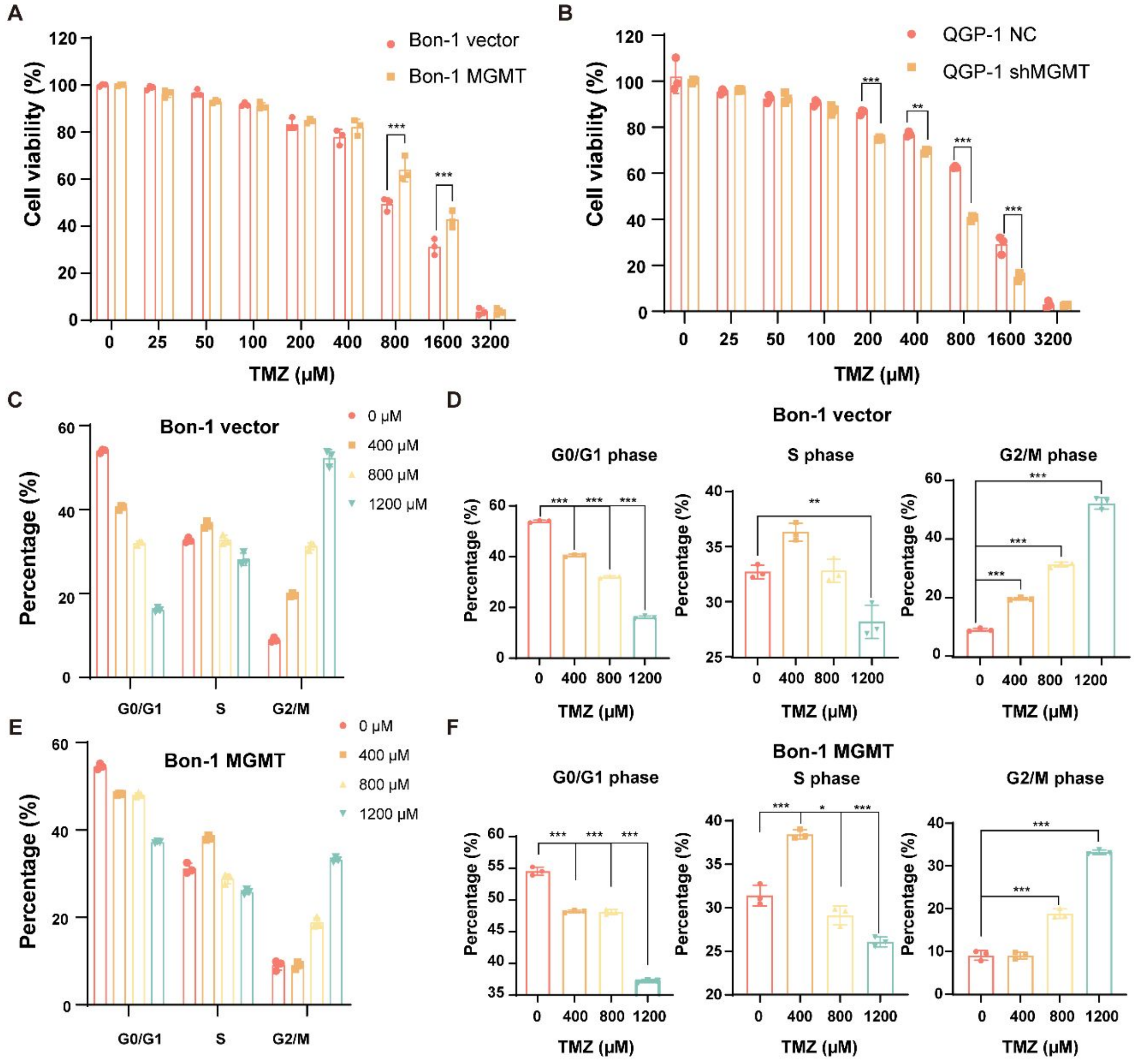

Figure S9. (A). Sensitivity of BON-1 vector and MGMT over-expressing cells to temozolomide (mean ± SD, two-way ANOVA with Sidak test, *** $\boldsymbol{P} < 0.001$, n = 3). (B). Sensitivity of QGP-1 NC and MGMT-knockdown cells to temozolomide (mean ± SD, two-way ANOVA with Sidak test, ** $\boldsymbol{P} < 0.01$, *** $\boldsymbol{P} < 0.001$, n = 3). (C). Overall changes in the cell cycle of BON-1 vector cells following temozolomide treatment (mean ± SD, n = 3). (D). Phase-specific changes in the cell cycle (G0/G1, S, G2/M) of BON-1 vector cells under temozolomide treatment (mean ± SD, one-way ANOVA with Dunnett's multiple comparison test, ** $\boldsymbol{P} < 0.01$, *** $\boldsymbol{P} < 0.001$, n = 3). (E). Overall cell cycle alterations in BON-1 over-expressing MGMT cells following temozolomide treatment (mean ± SD, n = 3). (F). Phase-specific cell cycle changes (G0/G1, S, G2/M) in BON-1 over-expressing MGMT cells under temozolomide treatment (mean ± SD, one-way ANOVA with Dunnett's multiple comparison test, * $\boldsymbol{P} < 0.05$, *** $\boldsymbol{P} < 0.001$, n = 3).

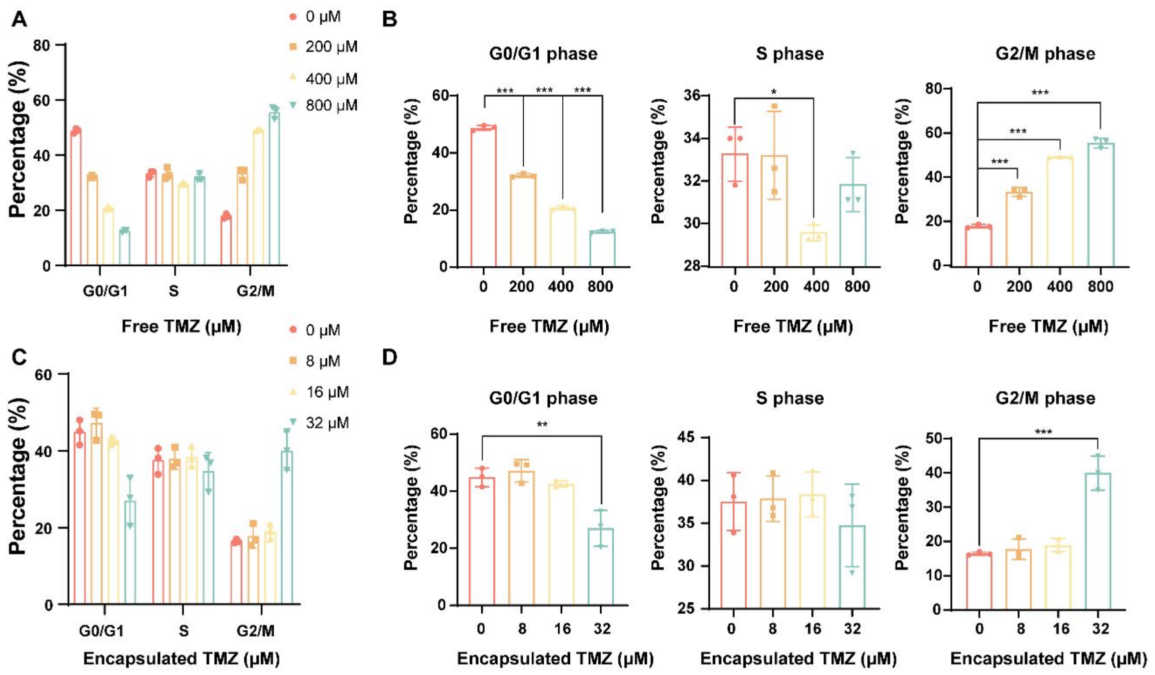

Figure S10. (A). Overall cell cycle changes in QGP-1 cells treated with free temozolomide (mean ± SD, n = 3). (B). Phase-specific changes in the cell cycle (G0/G1, S, G2/M) of QGP-1 cells under free temozolomide treatment (mean ± SD, one-way ANOVA with Dunnett's multiple comparison test, * ***P*** < 0.05, *** ***P*** < 0.001, n = 3). (C). Overall cell cycle changes in QGP-1 cells following encapsulated temozolomide treatment (mean ± SD, n = 3). (D). Phase-specific changes in the cell cycle (G0/G1, S, G2/M) of QGP-1 cells under encapsulated temozolomide treatment (mean ± SD, one-way ANOVA with Dunnett's multiple comparison test, ** ***P*** < 0.01, *** ***P*** < 0.001, n = 3).

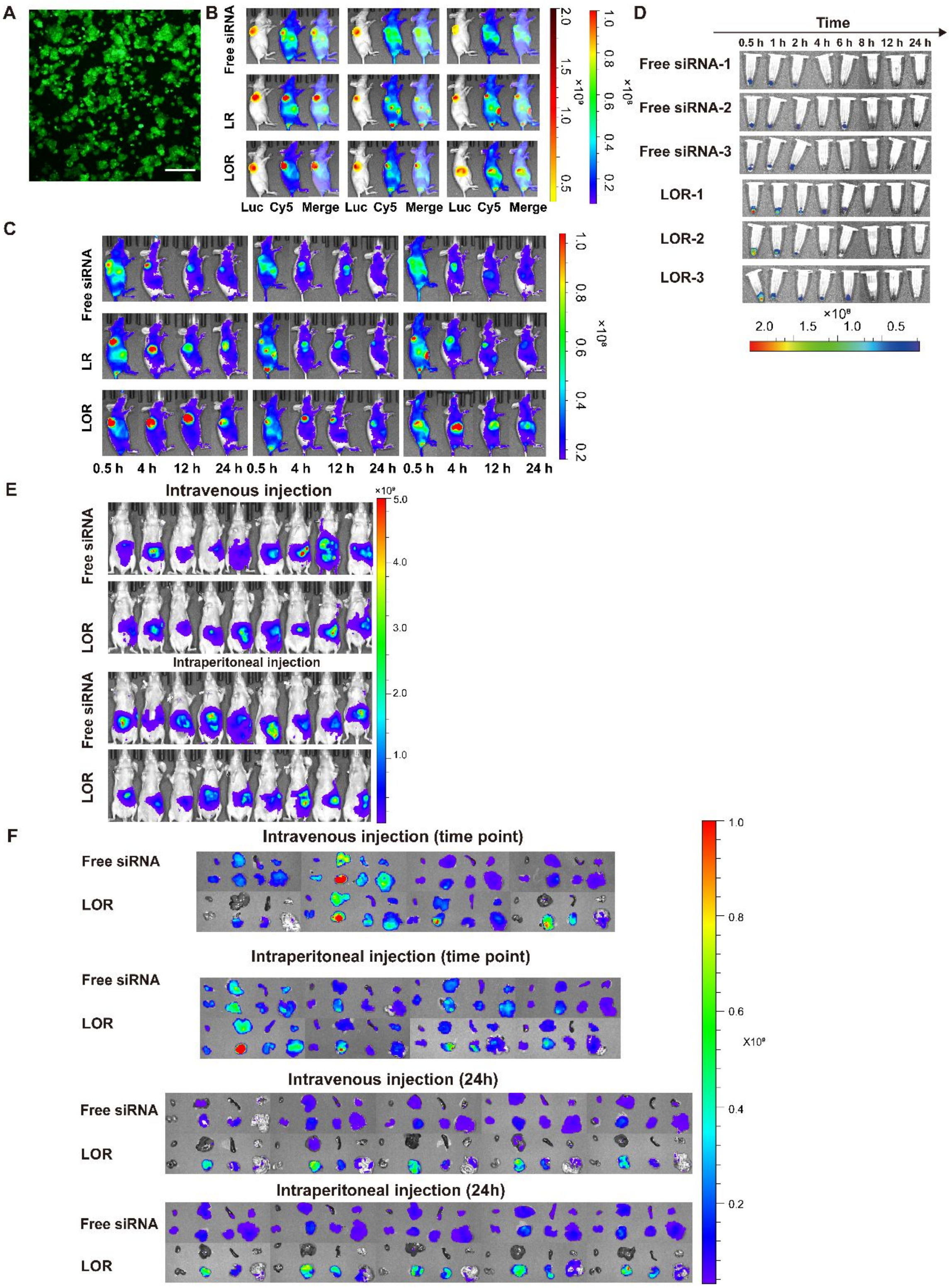

Figure S11. (A). Fluorescent microscopy of GFP-luc dual-labeled QGP-1 cells; scale bar represented 500 μm. (B) *In vivo* distribution of siRNA in mice bearing subcutaneous QGP-1 tumors, showing the highest tumor colocalization for LOR compared to LR and free siRNA. Bioluminescence (Luc) and fluorescence (Cy5) images were captured 0.5 hours post-injection. Color scale indicated radiance (p/sec/cm²/sr) ranges (left: min = 5.00×10^7, max = 2.00×10^9; right: min = 5.00×10^6, max = 1.00×10^8). (C) Time-

course fluorescence imaging demonstrating that LOR exhibited the longest circulation time compared to LR and free siRNA. Cy5 imaging was performed at 0.5-, 4-, 12-, and 24-hours post-injection. Color scale represented radiance (p/sec/cm²/sr) (min = 8.00 $\times 10^6$, max = 1.00 $\times 10^8$). (D) IVIS imaging of mouse blood samples from free siRNA and loaded siRNA treatment groups. Color scale referred to the range of radiant efficiency (p/sec/cm²/sr/µW/cm²) (min = 1.23 $\times 10^7$, max = 1.23 $\times 10^8$). (E) Bioluminescence (Luc) imaging of *in vivo* mice with orthotopic tumor models indicating the successful construction of orthotopic models. Color scale indicated radiance (p/sec/cm²/sr) ranges (left: min = 9.00 $\times 10^6$, max = 5.00 $\times 10^9$). (F) *Ex vivo* fluorescence imaging of harvested organs including heart, liver, spleen, lung, kidney, pancreas (tumor), stomach, intestine at 1-, 4-, 8-,12-, and 24-hours post-injection. Color scale represented radiant efficiency (p/sec/cm²/sr/µW/cm²) (organs, min = 3.00 $\times 10^7$, max = 1.00 $\times 10^9$). The order of organs: first row (left to right) heart, liver, spleen, lung; second row (left to right) kidney, pancreas, stomach, intestine.

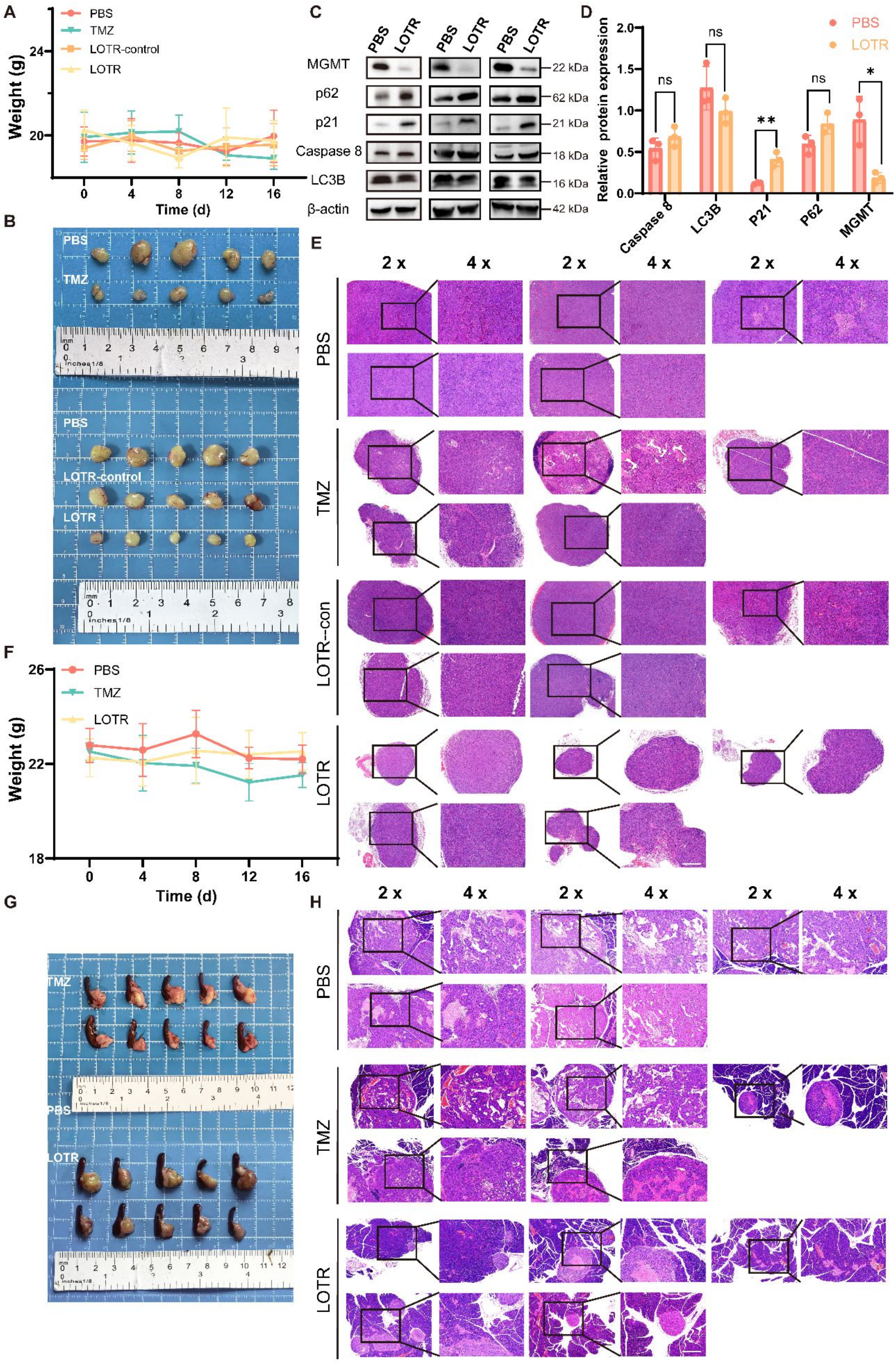

Figure S12. (A) Body weight analysis in mice with subcutaneous tumors (mean ± SD, two-way ANOVA with Sidak test, ***P*** = 0.20, n = 5). (B) Anatomical image of tumors

from the subcutaneous tumor model. (C) Western blot analysis of tumor tissues from subcutaneous tumor models treated with PBS and LOTR, showing specific protein expression changes. (D) Quantification of western blot analysis from subcutaneous tumor models treated with PBS and LOTR (* $\boldsymbol{P} < 0.05$, ** $\boldsymbol{P} < 0.01$, n = 3). (E). H&E staining of tumor sections from the subcutaneous model, scale bar represented 625 μm (4 × magnification). (F). Body weight analysis in mice with orthotopic pancreatic model (mean ± SD, two-way ANOVA with Sidak test, $\boldsymbol{P} = 0.58$, n = 5). (G). Anatomical image of tumors from the orthotopic pancreatic model. f. H&E staining of tumor sections from the orthotopic pancreatic model, scale bar represented 625 μm (4 × magnification).

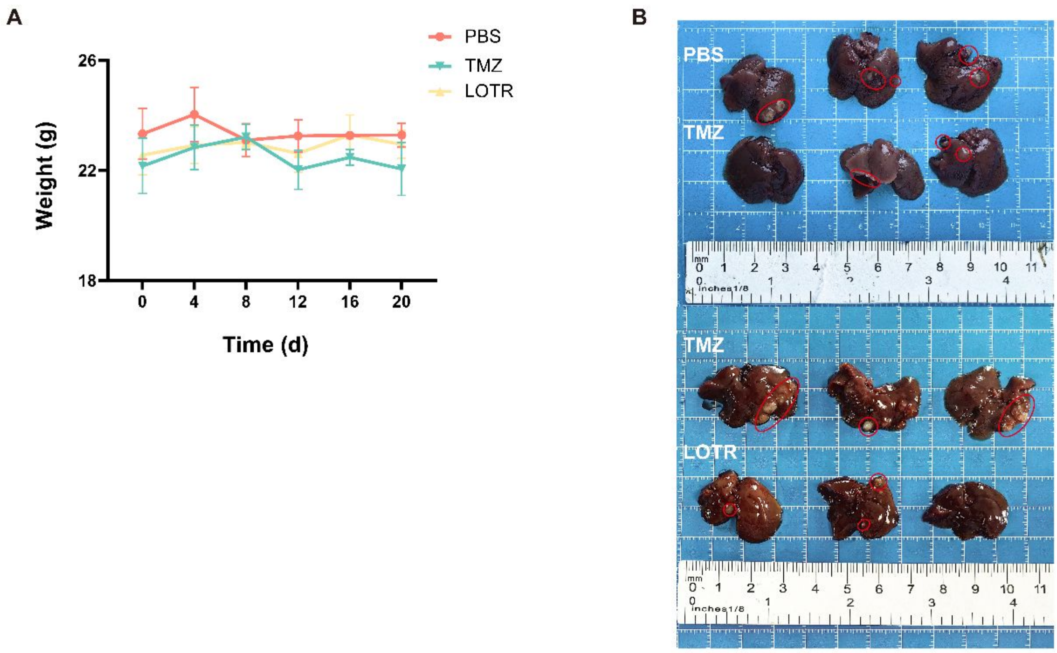

Figure S13. (A) Body weight analysis in mice with liver metastasis (mean ± SD, two-way ANOVA with Sidak test, ***P*** = 0.72, n = 3). (B) Anatomical image of tumors from the liver metastasis model.

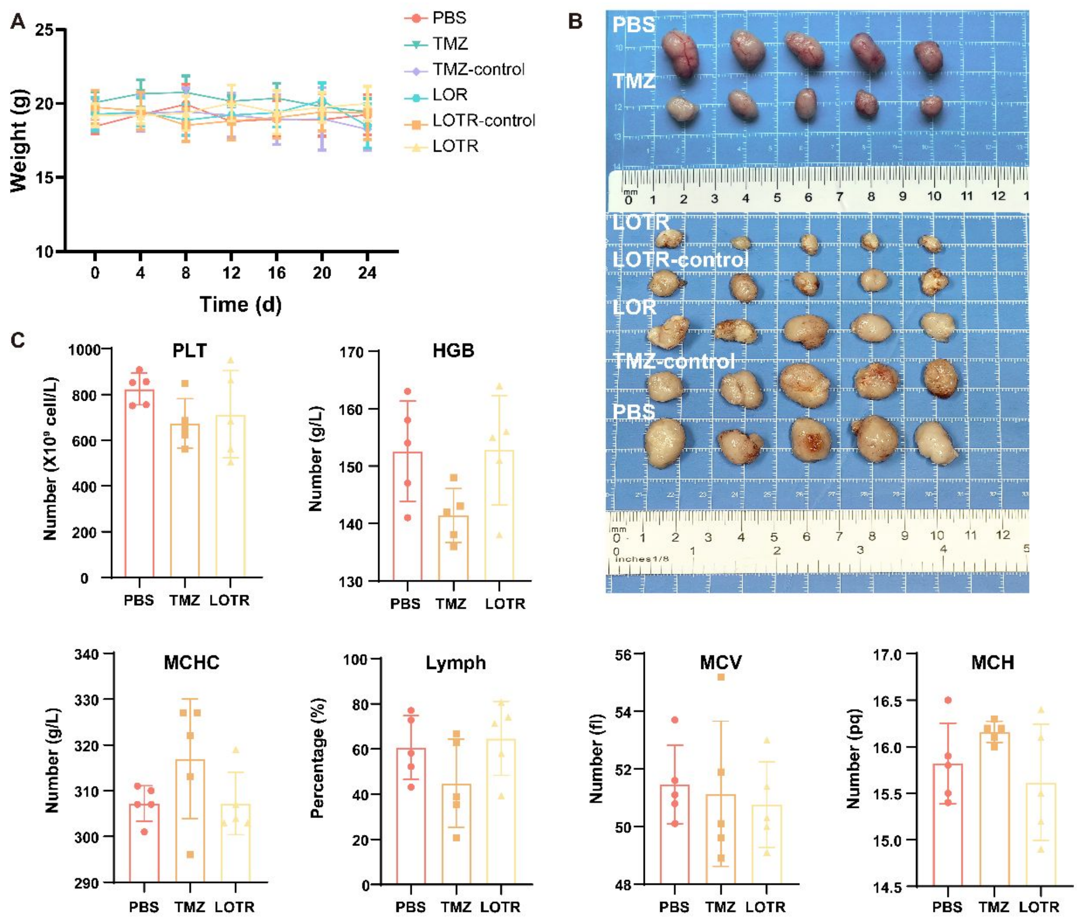

Figure S14. (A) Body weight analysis in PDX model (mean ± SD, two-way ANOVA with Sidak test, ***P*** = 0.82, n = 5). (B) Anatomical image of tumors from PDX model. (C) Complete blood count (CBC) analysis of blood samples from subcutaneous tumor models treated with PBS, TMZ, or LOTR (mean ± SD, one-way ANOVA with Dunnett's multiple comparison test, n = 5).

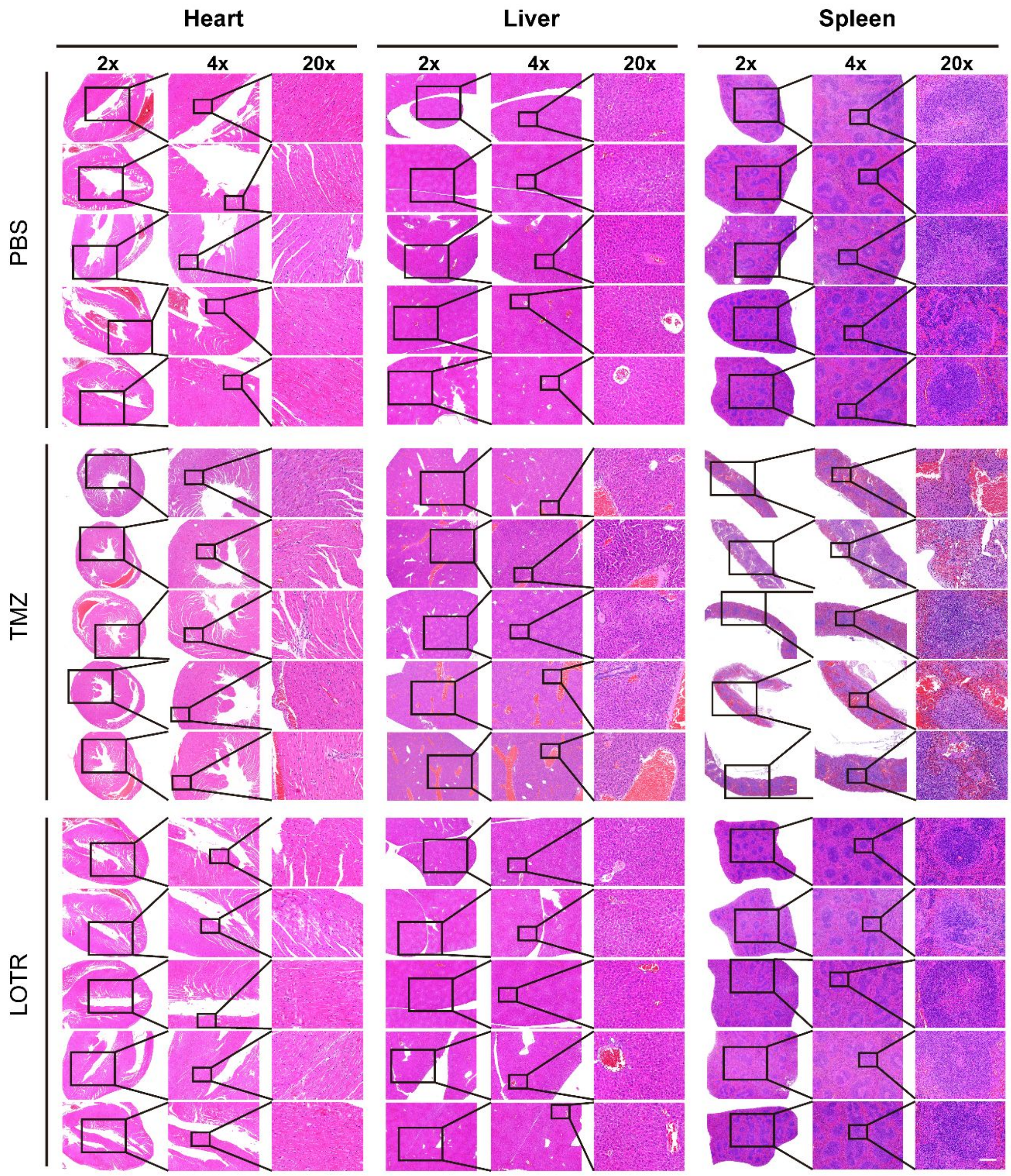

Figure S15. HE staining for organs (heart, liver, spleen) harvested from subcutaneous models, scale bar represented 100 μm (20× magnification).

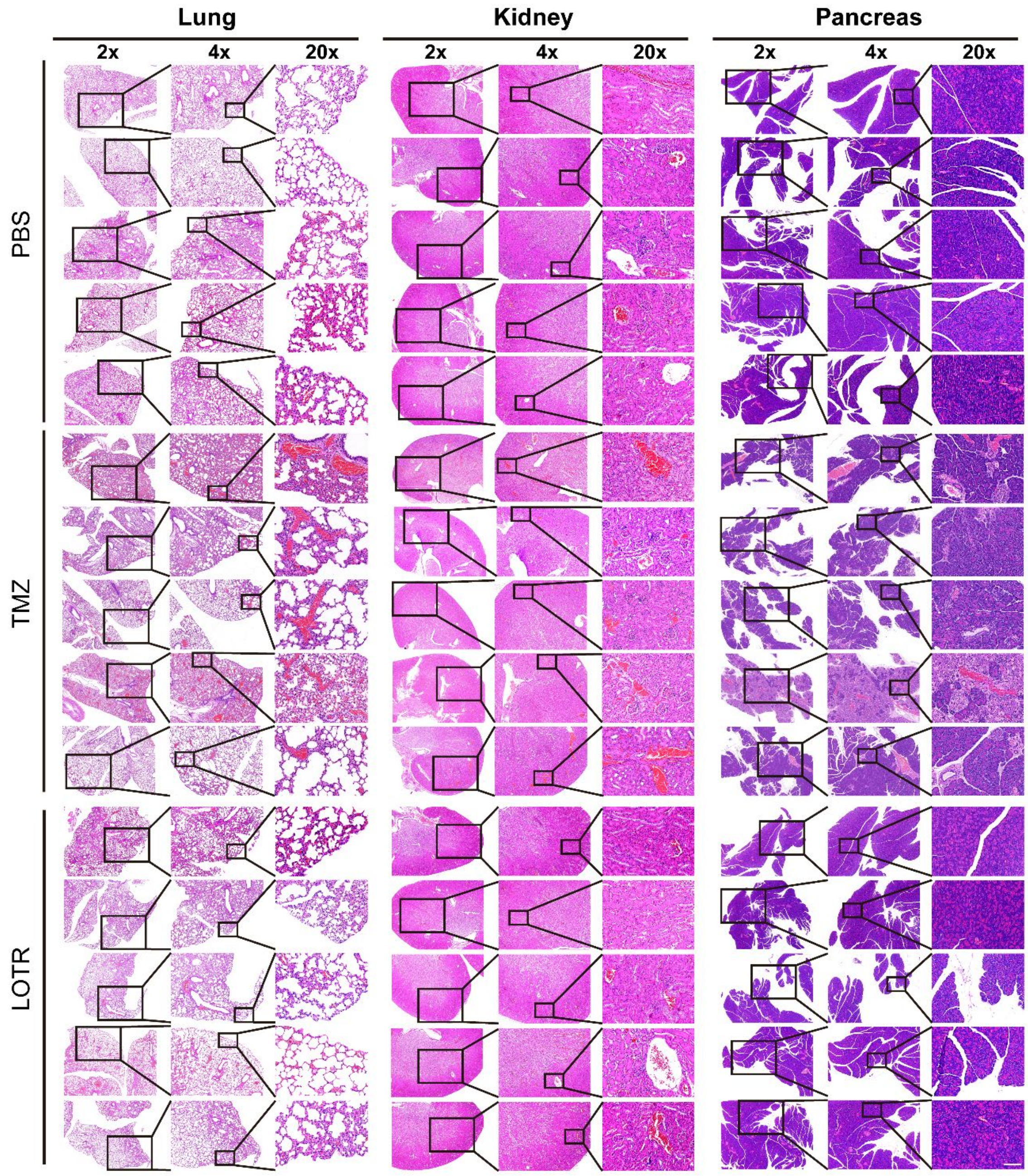

Figure S16. HE staining for organs (lung, kidney, pancreas) harvested from subcutaneous models, scale bar represented 100 μm (20× magnification).

Table S1. Quantitative organ distribution analysis in 24-hours post-injection.

	Free siRNA i.v		Free siRNA i.p		LOR i.v		LOR i.P	
	Mean	SD	Mean	SD	Mean	SD	Mean	SD
Pancreas(tumor)	1.50E+07	5.19E+06	1.99E+07	6.84E+06	2.39E+08	5.38E+07	3.51E+08	1.22E+08
Heart	9.73E+06	2.11E+06	8.73E+06	2.03E+06	5.56E+06	3.43E+06	3.81E+06	1.27E+05
Liver	9.08E+07	3.10E+07	8.85E+07	3.02E+07	1.17E+07	7.36E+06	1.19E+07	6.18E+06
Spleen	2.29E+07	1.05E+07	1.35E+07	5.07E+06	6.16E+06	1.47E+06	4.79E+06	8.67E+05
Lung	2.88E+07	1.18E+07	2.37E+07	5.36E+06	7.66E+06	3.39E+06	6.41E+06	1.22E+06
Kidney	5.64E+07	1.34E+07	5.35E+07	1.33E+07	1.04E+07	4.05E+06	6.14E+06	4.90E+05
Stomach	6.29E+07	9.28E+06	5.20E+07	1.50E+07	4.39E+07	1.10E+07	9.58E+07	1.27E+07
Intestine	6.82E+07	1.21E+07	8.81E+07	1.11E+07	1.74E+07	2.55E+06	2.43E+07	3.12E+06

*SD refers to standard deviation

Table S2. Details of dosages for each component used in murine models.

Group	Octreotide modification	Temozolomide	siRNA	Analytical objectives
PBS	/	/	/	Negative control
TMZ	/	40 mg/kg	/	Positive control
TMZ-control	/	0.88 mg/kg	/	Efficacy Control
LOR	Y	/	siMGMT,0.4 mg/kg	/
LOTR-control	Y	0.88 mg/kg	sicontrol,0.4 mg/kg	/
LOTR	Y	0.88 mg/kg	siMGMT,0.4 mg/kg	/

*Y refers to YES

Table S3. Abbreviations used in the text.

Abbreviation	Full Name
panNETs	pancreatic neuroendocrine tumors
TMZ	temozolomide
MGMT	O6-methylguanine-DNA methyltransferase
LNPs	Lipid nanoparticles
SSTRs	somatostatin receptors
OS	overall survival
ORR	objective response rate

PFS	progression-free survival
AEs	adverse events
RNAi	RNA interference
ESBO	epoxidized soybean oil
DLS	dynamic light scattering
TEM	Transmission electron microscopy
PDI	the polydispersity index
PBS	phosphate-buffered saline
EdU	5-ethynyl-2′-deoxyuridine
SSTR2	somatostatin receptor 2
IV	intravenous
IP	intraperitoneal
H&E	hematoxylin and eosin
IHC	immunohistochemistry
IVIS	*In vivo* imaging system
PET/CT	positron emission tomography / computed tomography
CBC	complete blood count
WBC	white blood cell
RBC	red blood cell
Neut	neutrophil
HCT	hematocrit
RDW	red cell distribution width
MPV	mean platelet volume
DMF	dimethylformamide
TEA	triethylamine
FBS	fetal bovine serum
FACS	fluorescence-activated cell sorting
SDS	sodium dodecyl sulfate
TAE	tris-acetate-EDTA
BCA	bicinchoninic acid
BSA	bovine serum albumin
PVDF	polyvinylidene fluoride
TBST	tris-buffered saline with Tween-20
ROS	reactive oxygen species
SD	standard deviation

Table S4. The components of the LNP delivery systems

LOTR	LNPs modified with Octreotide to co-deliver the TMZ and MGMT-siRNA
LOTR-control	LNPs modified with Octreotide to co-deliver the TMZ and control siRNA
LOR	LNPs modified with Octreotide to deliver MGMT siRNA
LOR-control	LNPs modified with Octreotide to deliver control siRNA
LR	LNPs to deliver MGMT siRNA
LR-control	LNPs to deliver control siRNA

Supporting Information references:

1. Tang, Z., Yu, F., Hsu, J.C., Shi, J., and Cai, W. (2024). Soybean Oil-Derived Lipids for Efficient mRNA Delivery. Adv Mater *36*, e2302901. 10.1002/adma.202302901.
2. Chen, Y., Huang, Y., Li, Q., Luo, Z., Zhang, Z., Huang, H., Sun, J., Zhang, L., Sun, R., Bain, D.J., et al. (2023). Targeting Xkr8 via nanoparticle-mediated in situ co-delivery of siRNA and chemotherapy drugs for cancer immunochemotherapy. Nat Nanotechnol *18*, 193-204. 10.1038/s41565-022-01266-2.
3. Soares, K.C., Foley, K., Olino, K., Leubner, A., Mayo, S.C., Jain, A., Jaffee, E., Schulick, R.D., Yoshimura, K., Edil, B., and Zheng, L. (2014). A preclinical murine model of hepatic metastases. J Vis Exp, 51677. 10.3791/51677.
